# Initiating Antiretroviral Therapy for HIV at a Patient’s First Clinic Visit: The RapIT Randomized Controlled Trial

**DOI:** 10.1371/journal.pmed.1002015

**Published:** 2016-05-10

**Authors:** Sydney Rosen, Mhairi Maskew, Matthew P. Fox, Cynthia Nyoni, Constance Mongwenyana, Given Malete, Ian Sanne, Dorah Bokaba, Celeste Sauls, Julia Rohr, Lawrence Long

**Affiliations:** 1 Department of Global Health, Boston University School of Public Health, Boston, Massachusetts, United States of America; 2 Health Economics and Epidemiology Research Office, Department of Internal Medicine, School of Clinical Medicine, Faculty of Health Sciences, University of the Witwatersrand, Johannesburg, South Africa; 3 Department of Epidemiology, Boston University School of Public Health, Boston, Massachusetts, United States of America; 4 Health Department, City of Johannesburg, Johannesburg, South Africa; Rwanda Ministry of Health, RWANDA

## Abstract

**Background:**

High rates of patient attrition from care between HIV testing and antiretroviral therapy (ART) initiation have been documented in sub-Saharan Africa, contributing to persistently low CD4 cell counts at treatment initiation. One reason for this is that starting ART in many countries is a lengthy and burdensome process, imposing long waits and multiple clinic visits on patients. We estimated the effect on uptake of ART and viral suppression of an accelerated initiation algorithm that allowed treatment-eligible patients to be dispensed their first supply of antiretroviral medications on the day of their first HIV-related clinic visit.

**Methods and Findings:**

RapIT (Rapid Initiation of Treatment) was an unblinded randomized controlled trial of single-visit ART initiation in two public sector clinics in South Africa, a primary health clinic (PHC) and a hospital-based HIV clinic. Adult (≥18 y old), non-pregnant patients receiving a positive HIV test or first treatment-eligible CD4 count were randomized to standard or rapid initiation. Patients in the rapid-initiation arm of the study (“rapid arm”) received a point-of-care (POC) CD4 count if needed; those who were ART-eligible received a POC tuberculosis (TB) test if symptomatic, POC blood tests, physical exam, education, counseling, and antiretroviral (ARV) dispensing. Patients in the standard-initiation arm of the study (“standard arm”) followed standard clinic procedures (three to five additional clinic visits over 2–4 wk prior to ARV dispensing). Follow up was by record review only. The primary outcome was viral suppression, defined as initiated, retained in care, and suppressed (≤400 copies/ml) within 10 mo of study enrollment. Secondary outcomes included initiation of ART ≤90 d of study enrollment, retention in care, time to ART initiation, patient-level predictors of primary outcomes, prevalence of TB symptoms, and the feasibility and acceptability of the intervention. A survival analysis was conducted comparing attrition from care after ART initiation between the groups among those who initiated within 90 d. Three hundred and seventy-seven patients were enrolled in the study between May 8, 2013 and August 29, 2014 (median CD4 count 210 cells/mm^3^). In the rapid arm, 119/187 patients (64%) initiated treatment and were virally suppressed at 10 mo, compared to 96/190 (51%) in the standard arm (relative risk [RR] 1.26 [1.05–1.50]). In the rapid arm 182/187 (97%) initiated ART ≤90 d, compared to 136/190 (72%) in the standard arm (RR 1.36, 95% confidence interval [CI], 1.24–1.49). Among 318 patients who did initiate ART within 90 d, the hazard of attrition within the first 10 mo did not differ between the treatment arms (hazard ratio [HR] 1.06; 95% CI 0.61–1.84). The study was limited by the small number of sites and small sample size, and the generalizability of the results to other settings and to non-research conditions is uncertain.

**Conclusions:**

Offering single-visit ART initiation to adult patients in South Africa increased uptake of ART by 36% and viral suppression by 26%. This intervention should be considered for adoption in the public sector in Africa.

**Trial Registration:**

ClinicalTrials.gov NCT01710397, and South African National Clinical Trials Register DOH-27-0213-4177.

## Abstract 2

In the RapIT randomized controlled trial, Sydney Rosen and colleagues investigate whether accelerated initiation of antiretroviral therapy can improve viral suppression for HIV patients in South Africa.

## Author Summary

**Why Was This Study Done?:**

- One of the most persistent operational challenges facing antiretroviral therapy (ART) programs for HIV/AIDS in sub-Saharan Africa is late presentation of patients for care and high rates of attrition from care between HIV testing and ART initiation.
- One reason for this is that starting ART in many countries is a lengthy and burdensome process, imposing long waits and multiple clinic visits on patients; in South Africa, the country with the world’s largest HIV treatment program, patients must typically make five or six clinic visits, starting with an HIV test, before they receive medications.
- There have not yet been any controlled evaluations of an integrated, rapid HIV treatment initiation algorithm that allows patients to initiate ART in a single clinic visit, so the RapIT trial was done to find out if “same-day initiation” of ART would increase the number of patients starting treatment and improve overall health outcomes, compared to current practices.

**What Did the Researchers Do and Find?:**

- We randomly assigned 377 adult patients at two public clinics in Johannesburg, South Africa, who had provided consent to participate in the study to one of two groups.
- Patients in the group assigned to receive rapid treatment initiation were offered the chance to start treatment on the same day as their first clinic visit, using rapid, point-of-care laboratory tests and an accelerated sequence of other steps, including a physical exam, education, and counseling.
- Patients in the group assigned to receive standard treatment initiation followed the standard schedule for treatment initiation used by the clinics, which usually required three to five additional clinic visits over a 2–4 wk period.
- After the study enrollment visit, patients were followed up by reviewing their regular clinic medical records, to determine how many did start treatment and how many were still in care and had good outcomes, as indicated by a suppressed viral load, 10 mo later.
- We found that 97% of patients in the rapid initiation group had started ART by 90 d after study enrollment—three-quarters of them on the same day—compared to 72% of patients in the standard initiation group.
- By 10 mo after study enrollment, 64% of patients in the rapid group had good outcomes compared to 51% in the standard group.
- Rapid initiation group patients spent roughly two and a half hours in the clinic to complete all the steps required before they got their medications.

**What Do These Findings Mean?:**

- The RapIT (Rapid Initiation of Treatment) trial showed that it is possible to initiate nearly all eligible patients on HIV therapy, and to do so in a much shorter time interval than previously required.
- By showing that offering the opportunity to start treatment on the spot, without delay, overcomes many barriers patients would otherwise face, this study demonstrates that same-day ART initiation is an effective strategy for improving health outcomes.
- More patients in the rapid initiation group dropped out of care after starting treatment than in the standard initiation group; although the rapid initiation group still had better health outcomes overall, adherence support after starting treatment remains essential.
- The findings of this study are limited because the study took place in only two clinics in one part of South Africa and was carried out by study staff, not by regular clinic staff.
- Based on this study’s results, consideration could be given to accelerating the process of ART initiation in many different settings and for different types of patients.

## Introduction

One of the most persistent operational challenges facing antiretroviral therapy (ART) programs for HIV/AIDS in sub-Saharan Africa is late presentation of patients for care and high rates of attrition from care between HIV testing and ART initiation, with baseline median CD4 cell counts remaining well below 200 cells/mm^3^ in the region despite steadily rising eligibility thresholds [1]. Even among those who have been diagnosed and found to be treatment-eligible, loss to care before starting ART has consistently been estimated at a third to a quarter of patients [2,3]. While many of those who drop out of care prior to ART initiation will make their way back at a later time, they will almost certainly have lower CD4 counts and more symptoms of illness than when they first tested positive. Some will be very sick or die before treatment can be started, and those who do eventually start will have a poorer prognosis on treatment than if they had begun treatment earlier [4,5]. Offering ART to all who test positive regardless of CD4 count, as is now recommended by the World Health Organization [6], will make little difference if those who test positive fail to initiate treatment.

There are likely many causes of loss to care before treatment initiation, but one reason observed is that starting ART in many countries is a lengthy and burdensome process, requiring long waits and multiple clinic visits [7,8]. In South Africa, the country with the world’s largest HIV treatment program [9], the process typically includes an HIV test (visit 1), determination of treatment eligibility (visit 2), adherence education and counseling and baseline blood tests (visits 3, 4, and 5), and physical examination and dispensing of antiretrovirals (ARVs) (visit 6). The proliferation of visits has three main causes. First, clinic receipt of printed test results from centralized laboratories typically takes several days, if not longer. Second, a belief remains that to ensure adherence, patients must participate in multiple preparatory educational and counseling sessions [2,10,11]. And third, clinics have had little motivation to accelerate the initiation process for patients who are not critically ill, as standard performance indicators do not include the proportion of eligible patients who actually initiate ART, nor the time required to do so.

If patients are deterred from starting treatment by the complexity of the process, then one strategy for reducing loss of patients prior to ART initiation and encouraging earlier treatment initiation may be to shorten the time period, reduce the number of visits, and simplify the steps required before medications are dispensed. This strategy depends critically on two factors: a clinic’s willingness and ability to adjust its schedules and procedures to compress and accelerate the required steps, and the availability of rapid, point-of care (POC) laboratory assays that eliminate delays in receiving whatever lab results are required for initiation. There have not yet been any rigorous, controlled evaluations of an integrated, rapid HIV treatment initiation algorithm incorporating procedural changes and POC tests for adult, non-pregnant patients. We therefore conducted a randomized controlled trial of rapid ART initiation that allowed patients in public sector clinics in Johannesburg, South Africa to have treatment eligibility determined, all treatment preparation steps performed, and ARV medications dispensed on the day of their first HIV-related clinic visit.

## Methods

RapIT (Rapid Initiation of Treatment) was an unblinded, individually randomized, controlled trial of a service delivery intervention. It was approved by the Institutional Review Board of Boston University Medical Campus (H-31880) and the Human Research Ethics Committee (Medical) of the University of the Witwatersrand (M120843) and is registered with ClinicalTrials.gov, number NCT01710397.

### Study Sites, Infrastructure, and Staffing

RapIT was conducted at two public sector outpatient clinics. Site 1 is a primary health clinic serving an urban informal settlement population on the edge of Johannesburg. Site 2 is a large, hospital-based HIV clinic serving an urban formal and informal population within Johannesburg. Both sites follow South African national treatment guidelines for ART initiation, ARV regimens, and monitoring [12]. During the period of study enrollment, May 8, 2013–August 29, 2014, the prevailing threshold for ART eligibility was a CD4 count ≤ 350 cells/mm^3^ or a WHO Stage 3/4 clinical condition. Requirements for care prior to initiating ART are not standardized in South Africa [13], but both sites generally required four to five clinic visits between HIV testing and dispensing the first month’s supply of ARVs.

At each site, a small clinic room with security bars, running water, and basic furnishings was designated for study equipment and supplies, POC instruments, and files. As all the POC instruments were designed as desktop devices, no separate laboratory was needed. An outdoor booth for safe collection of sputum samples from tuberculosis (TB) suspects was constructed at Site 1 and made available for both study arms; existing facilities for this purpose were used at Site 2. Clinical procedures were performed by study nurses with the same level of clinical certification as existing primary health care nurses at the sites. Non-clinical procedures (consent, questionnaire, education, counseling, patient flow management) were implemented by study assistants with qualifications comparable to those of experienced lay counselors at the sites. All study staff received study and instrument-specific training. A small stipend (R1000/month, equivalent to US$86 at the exchange rate at the time of the study) was paid to clinic lay counselors at Site 1 and a messenger at Site 2 who assisted by referring potential study participants to the study assistant.

### Study Population

The study enrolled adult (≥18 y old), non-pregnant patients who presented to have an HIV test, provide a blood sample for a CD4 count if already known to be HIV-infected, or receive the results of the patient’s first treatment-eligible CD4 count. During pre-screening and screening, patients who had previously been found to be eligible for ART, were already on ART or reported receiving it in the past 12 mo, indicated that they intended to seek HIV care during the next 12 mo at a different clinic, were judged by clinic or study staff to be physically or emotionally unable to provide consent or participate in all study procedures, or did not meet other study inclusion criteria were excluded. Potential participants whose visit purpose was to have an HIV test were enrolled; those found post-enrollment not to be eligible for ART were subsequently withdrawn upon determination of ineligibility. Potential participants whose visit purpose was to receive a CD4 count result and were not eligible for treatment on the basis of that CD4 count were not enrolled.

Participants were individually randomized 1:1 to either rapid treatment initiation or standard-of-care treatment initiation, using block randomization in blocks of 6. Sealed, opaque envelopes containing the allocations were prepared by the local principal investigator and numbered sequentially. The envelopes were kept in sequential, numbered order at the study sites. After obtaining written informed consent, the study assistant opened the next sequentially numbered envelope to reveal the allocation.

### Study Design and Procedures

**Fig 1:**
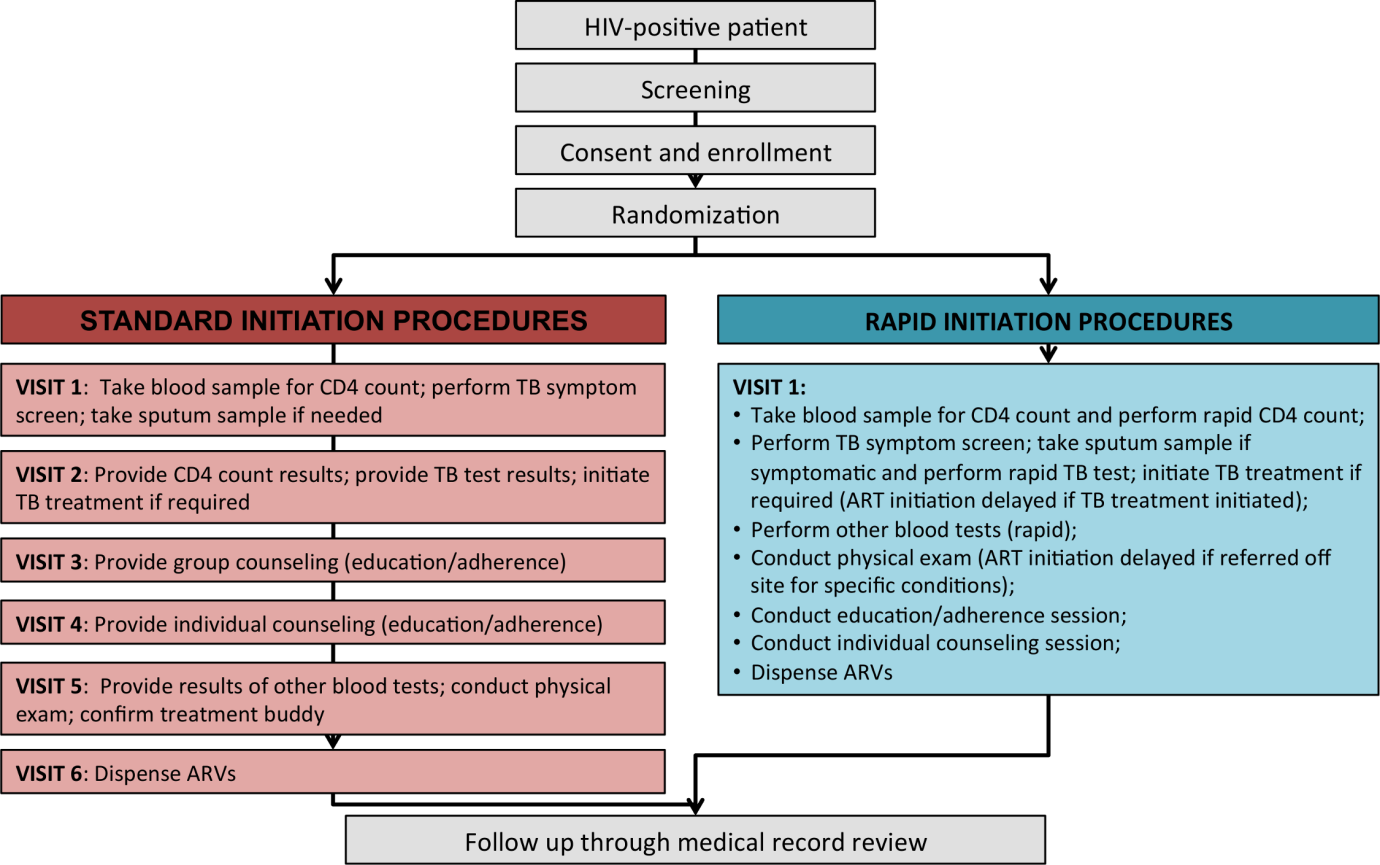
Standard initiation of treatment and rapid initiation procedures and visit schedule.

Procedures for each study arm are illustrated in Fig 1. Standard-of-care treatment initiation followed existing procedures at the sites as closely as possible. Study staff interaction with participants was limited to screening for study eligibility, obtaining written informed consent, administering a questionnaire, and referring patients to clinic staff for either a blood draw for a CD4 count or a next visit appointment if the patient already had results of a CD4 count in hand. After referral, patients in the standard-initiation arm of the study were followed passively, through medical record review, and had no further interaction with the study. Standard-of-care procedures for ART initiation at both study sites included a CD4 count to determine eligibility, TB symptom screening followed by a TB test and TB treatment initiation if required, pre-initiation blood tests (hemoglobin, creatinine, and alanine aminotransferase (ALT)), group and individual counseling and education sessions, and a physical examination. All samples for laboratory tests were sent to centralized public sector laboratories, requiring patients to make separate clinic visits to provide biological samples and to receive results. Once ART eligibility was determined, initiation typically required three to four more clinic visits over a period of 2–4 wk. Patients who were very ill or found to have low CD4 counts could be “fast-tracked,” with the schedule shown in Fig 1 completed in as little as one week.

For patients randomized to rapid initiation, all the same procedures were performed, but the use of a compressed and accelerated schedule and rapid laboratory instruments at point of care allowed them all to be completed in a single visit (Box 1). Patients offered rapid initiation typically completed each step in order, with little or no waiting time in between unless a TB test was required, which entailed a wait to process the sample. Patients who enrolled in the study too late in the day for all steps to be completed before the clinic closed were asked to return the next day to finish study procedures. Patients who were randomized to rapid initiation but did not have time to participate on the day of enrollment or wished to delay for other reasons were given up to 30 d to return and be initiated under rapid procedures. Those returning beyond 30 d were offered standard initiation by the clinic.

**Box 1. Rapid Initiation Procedures**

**CD4 count**

Patients who enrolled in the study and did not already have CD4 count results from a test performed within the previous 6 mo were given a rapid CD4 count using the Alere Pima CD4 Test (http://alerehiv.com/hiv-monitoring/alere-pima-cd4/) with venous blood draw. This test, previously evaluated in several studies in Africa [14–18], provides a CD4 count result from a capillary or venous blood sample in 20 min. Following the test, patients with a CD4 count ≤ 350 cells mm^3^ or evident physical symptoms or complaints that suggested a Stage 3 or 4 condition continued with study procedures. Those not eligible for ART were withdrawn from the study at this point and referred to the clinic for standard pre-ART monitoring.

**TB symptom screen and test**

While awaiting CD4 count results, a TB symptom screen was administered using South Africa’s four-question screening tool. All patients who reported symptoms were then asked to provide a sputum sample, which was immediately processed using the Cepheid Xpert MTB/RIF test (http://www.cepheid.com/us/cepheid-solutions/clinical-ivd-tests/critical-infectious-diseases/xpert-mtb-rif). This is the technology currently used for TB diagnosis in the public sector throughout South Africa, but it is located in centralized laboratories rather than at point of care [19]. It generates a TB diagnosis in 90 min [20]. Two sputum samples were run simultaneously to increase the reliability of results. Any patient who received a positive Xpert test was escorted to the clinic TB nurse to initiate TB treatment, which under national guidelines required a delay of at least 2 wk before ART could be initiated. Patients initiated on TB treatment were asked to return 2 wk later to complete rapid ART initiation on a second visit.

**Baseline tests**

Once eligibility for ART was established, pre-initiation blood tests (hemoglobin, creatinine, and ALT) were run on a point-of-care Reflotron Plus instrument (Roche, http://www.roche-diagnostics.co.in/Products/Pages/ReflotronPlusDry.aspx)[14] using the same blood sample dawn for the CD4 count. This instrument takes approximately 2 min to complete each test. A standard clinic urine dipstick pregnancy test was also conducted for female patients of child-bearing age.

**Physical exam**

A standard physical examination was conducted by the study nurse to identify any specific conditions or concerns prior to initiating ART. Initiation was delayed in patients found to have conditions that required referral to a hospital or consultation with the clinic’s doctor.

**Education session**

A condensed version of HIV/ART/adherence education was developed using the study clinics’ materials and provided to study participants. It was delivered in a one-on-one session by the study counselor in approximately 20 min.

**Counseling session**

After completing all tests, physical examination, and education session, each patient met individually with the study nurse, who reviewed results with the patient and provided an opportunity for the patient to ask any remaining questions and confirm that she or he was indeed ready for treatment initiation.

**Dispensing of ARVs**

The study nurses, like other qualified nurses in South Africa, were authorized to write prescriptions for ARVs, which could then be filled directly by the nurse from study room stock (Site 1) or at the on-site clinic pharmacy (Site 2). Study patients at Site 2 were served at the pharmacy immediately, rather than being required to wait in pharmacy queues to fill prescriptions. Once the initial 4 wk supply of ARVs was dispensed, study interaction with rapid group patients ceased. Patients were asked to return to the clinic for monitoring and prescription refill by clinic staff in 1 mo, consistent with routine practice.

After the enrollment visit, or completion of rapid initiation procedures for patients in the rapid-initiation arm of the study (“rapid arm”) who delayed initiation but returned to complete it within 30 d, the study team had no further contact with study patients. Patients who started ART in either arm received standard-of care treatment management from the clinic, which called for monitoring visits and medication refills at 1, 2, 3, 6, and 12 mo after initiation, with a routine viral load test at the 6 mo visit.

### Outcomes and Data

The primary, protocol-defined outcome for the study was viral suppression (≤400 copies/ml) within 10 mo of study enrollment, a time period selected to capture the 6 mo routine monitoring visit called for by national guidelines. Ten months was selected as the endpoint to allow patients to take up to 3 mo to initiate ART and to be up to 1 mo late for the 6 mo routine visit. Because the study sites occasionally omitted the 6 mo viral load and performed the test only at 12 mo, we considered a patient with a suppressed viral load test result any time from 3 to 12 mo after study enrollment to have achieved viral suppression. In this analysis, missing viral load test results were regarded as failures; only patients with recorded, suppressed viral load results were defined as virally suppressed. To account for the possibility that viral load results could be missing due to clinic oversight in not ordering the test, rather than patient default, and to investigate the possibility that rapid initiation merely shifts attrition from before to after treatment initiation, we also report the secondary outcome of retention in care at 10 mo after study enrollment, with retention defined as any HIV-related clinic visit in months 5–10 after study enrollment, regardless of viral load.

Although viral suppression was the primary outcome assessed, the pathway by which the study aimed to increase suppression was reduction of attrition between HIV testing and treatment initiation. We therefore report initiation of treatment within 90 d of study enrollment as a secondary outcome, with initiation defined as being dispensed a first month’s supply of ARVs. We also report uptake of treatment within 180 d, as a CD4 count result is considered to be valid under South African guidelines for 6 mo—after that, a patient must have a new CD4 count to establish eligibility for ART. Finally, we report the distribution of time (d) to treatment initiation in each group.

Other secondary outcomes evaluated in the study included the feasibility of the intervention, as indicated by the ability of both study sites to implement the accelerated algorithm; acceptability of the intervention, as measured by the proportion of patients offered rapid initiation who accepted it; patient-level predictors of the primary outcome; and, in the rapid arm, the prevalence of TB symptoms and confirmed TB disease and ART initiation among patients with TB.

After the enrollment visit, all data collection for both groups was by passive medical record review. Both study sites routinely utilized an electronic medical record system called TherapyEdge-HIV, into which patient data were entered retrospectively by data clerks from paper files (Site 1) or by a combination of clinicians in real time and data clerks from paper files (Site 2)[21]. This record system improved the completeness of the follow-up dataset used in the study. In instances of incomplete follow-up data—for example, if the database reported a clinic visit 6 mo after ART initiation but contained no viral load test result—study staff searched the clinics’ paper files and registers and the online data portal of the National Health Laboratory Service to determine if any additional information existed but had not been recorded in the clinics’ databases. The study team had no further contact with study participants after the enrollment visit so as not to have any influence on retention in care, a study outcome.

### Data Analysis

We designed the study to detect a 20% difference in viral suppression rates between the arms at 10 mo after study enrollment. With an α of 0.05, power of 90%, 1:1 randomization, and an uncorrected Fisher’s exact test, we estimated that we would need to enroll at least 124 HIV positive ART-eligible participants per group (248 total). We increased this to a maximum of 200 per group (400 total) to allow for stratification by site, sex, or age.

Characteristics at study enrollment of all randomized participants who met ART initiation and study inclusion criteria were summarized using simple proportions and medians with interquartile ranges (IQR) stratified by treatment arm. For the remaining analyses, we excluded patients who were found after randomization not to be eligible for ART or not to meet study inclusion criteria. We compared the proportions of patients achieving each dichotomized study outcome and present crude risk ratios (RR) and risk differences (RD) with 95% confidence intervals (CI) stratified by group. Baseline predictors of outcomes that appeared imbalanced by treatment arm were also adjusted for using log-linear regression models to estimate adjusted risk ratios (aRR). We estimated time to treatment initiation in days using a cumulative incidence curve. To investigate whether attrition after initiation of ART differed between the study arms, we performed a survival analysis comparing attrition from care after ART initiation among those who initiated within 90 d between the groups. Person-time accrued from ART initiation date to the earliest of loss to follow up, transfer, or 10 mo of follow up, and hazard ratios of attrition from care were estimated with Cox proportional hazards models. A stratified analysis was performed to detect effect measure modification by site or patient-level factors. Finally, to confirm that no imbalance was created by excluding patients after randomization for reasons other than ineligibility for ART or evidence of a previous eligible CD4 count, we conducted sensitivity analysis incorporating the excluded patients and assigning each a negative outcome.

## Results

**Fig 2:**
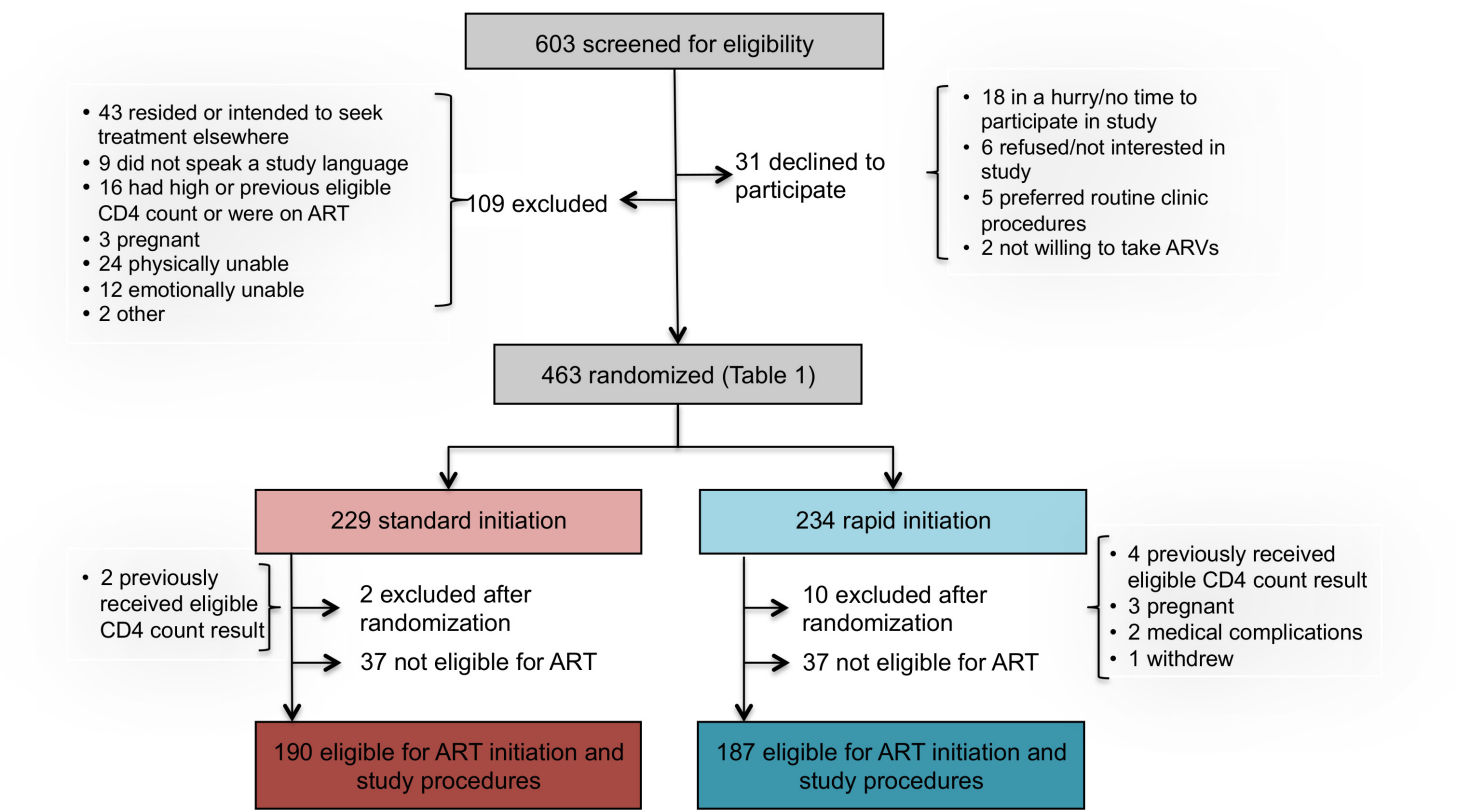
Study enrollment and randomization.

Between May 8, 2013, and August 29, 2014, 603 patients were screened for study eligibility and 463 provided written informed consent and were enrolled in the study (Fig 2). Of the 140 screened but excluded prior to randomization, 109 did not meet study eligibility criteria, including 43 who resided outside study clinic catchment areas or intended to seek further care elsewhere; 24 who were determined by the study assistant to be too ill for consent and study procedures; 16 who were not eligible on the basis of a prior CD4 count, were ineligible for ART, or were already on ART; 12 who were determined by the study assistant to be too emotionally upset to provide consent; 9 who did not speak any of the languages spoken by the study team; 3 who were found to be pregnant; and 2 who were excluded for other reasons. An additional 31 patients refused participation; of these, 18 were in a hurry and did not have time for study procedures, six did not wish to participate in the study, five stated that they would prefer standard care, and two were not willing to initiate therapy. Follow-up ended 10 mo after the last patient was enrolled (June 28, 2015).

Characteristics of patients in each study arm at time of enrollment are reported in Table 1. There were no important differences between the study arms in the variables shown. Just over half the participants were female and the median age was 35 y. The median CD4 count was less than 200 cells/mm^3^. Age, sex, and CD4 count characteristics of the study sample were similar to those of the overall non-pregnant patient populations initiating ART at the study clinics in 2014.

**Table 1:** Baseline characteristics of study sample (*n* = 463).

Variable	Standard arm	Rapid arm
*n* (randomized participants)	229	234
Enrollment site (*n*)		
Site 1 (primary health clinic)	124	126
Site 2 (hospital-based HIV clinic)	105	108
Age (median, IQR)	35.8 (29.5–41.6)	34.2 (29.0–40.1)
Sex (% female)	132 (58%)	129 (55%)
CD4 count (cells/mm^3^) (median, IQR)	195 (103–322)	224 (128–327)
Purpose of clinic visit (%)		
Have HIV test (diagnosed today)	100 (44%)	90 (38%)
Provide blood sample for CD4 count	8 (4%)	10 (4%)
Receive first CD4 count results	109 (47%)	112 (48%)
Other	11 (5%)	22 (10%)
Reason for treatment eligibility (%)		
CD4 count below threshold	182 (79%)	183 (78%)
Clinical condition Stage 3 or 4	3 (1%)	4 (2%)
Excluded (not eligible for treatment or study)	44 (20%)	47 (20%)
Household composition		
Live alone (% yes)	36 (16%)	41 (18%)
# other persons in house (median, IQR)	2 (1–4)	2 (1–3)
Household type (%)		
Formal house or flat	146 (63%)	165 (71%)
Informal dwelling or shack	83 (37%)	69 (29%)
Travel time to clinic (minutes) (median, IQR)	18 (9–24)	15 (9–27)
Employment status (%)		
Employed formally	68 (30%)	90 (38%)
Work informally	62 (27%)	54 (23%)
Unemployed, seeking work	91 (40%)	84 (36%)
Unemployed, not seeking work	8 (3%)	6 (3%)
Marital status (%)		
Married or long-term partner	173 (76%)	157 (67%)
Single, no long-term partner	41 (18%)	57 (24%)
Other (widowed, divorced)	15 (6%)	20 (9%)

Reasons for excluding patients during the study screening process are reported in Fig 2. The 603 patients screened represent a subset of those pre-screened by clinic counselors and then referred to the study assistant for screening. While pre-screening data, which were collected by the counselors and not by study staff, are of uncertain quality, they do provide some indication of the proportion of all patients presenting at clinics who could be eligible for rapid initiation. At Site 1, for which the pre-screening data are more complete, a total of 2,636 patients presenting at the clinic’s HIV counseling and testing service were pre-screened. More than half of these were HIV-negative (1,468/2,636, 56%) or known to have CD4 counts above the eligibility threshold or already on ART (114/2,636, 4%). Of the remaining 1,054, 325 (31%) were referred for study screening. Another 293/1,054 (28%) were judged by the counselors not to meet study protocol eligibility criteria (age, residence location, language, not first CD4 count) but would likely have been eligible for the intervention if it were offered as routine care. A fifth (225/1,054, 21%) were regarded by the counselors as too sick for study participation (not necessarily for ART initiation) and were referred to a clinic doctor or nurse for immediate care; it is not clear if they would have been eligible for the intervention or not. The remainder (20%) included patients who refused study participation (36/1,054, 3%) or refused any further care (12/1,054, 1%), were deemed too upset or emotionally distressed to participate (25/1,054, 2%), were referred directly to the clinic’s HIV or TB nurse rather than the study assistant (75/1,054, 7%), or were in a hurry or had no reason stated (63/1,254, 6%).

Among 463 patients screened and found eligible for study participation, 234 patients were randomized to rapid initiation and 229 to standard initiation (Fig 2). Upon completion of a CD4 count, which occurred after randomization for those who did not already have one in hand, 37 patients in each group were determined not to be eligible for ART under South African guidelines and were excluded from further data collection and from the analysis. An additional 12 patients were excluded after randomization, for reasons indicated in Fig 2. One hundred and ninety patients in the standard group and 187 in the rapid group (*n* = 377 total) were offered full study procedures and are included in the analysis below, with sensitivity analysis incorporating the six who were excluded after randomization for a reason other than ineligibility for ART or evidence of a prior eligible CD4 count.

The protocol-defined primary outcome for the study was viral suppression within 10 mo of study enrollment. As presented in Table 2, viral suppression by 10 mo was 64% (119/187) in the rapid arm and 51% (96/190) in the standard arm, indicating a risk difference of 13% (3%–33%) and a crude relative risk of 1.26 (1.05–1.50).

**Table 2:** ART initiation, 10-mo retention in care, and 10-mo viral suppression.

Outcome	Standard arm(%)*n* = 190	Rapid arm(%)*n* = 187	Crude risk difference(95% CI)	Crude relative risk(95% CI)
Initiated ≤ 90 d and suppressed by 10 mo (primary outcome)	96 (51%)	119 (64%)	13% (3%–23%)	1.26 (1.05–1.50)
*Of those* *not* *initiated ≤ 90 d and suppressed by 10 mo*	*94 (49%)*	*68 (36%)*		
* Not initiated*	*54 (28%)*	*5 (3%)*		
* Initiated but not suppressed*	*40 (21%)*	*63 (34%)*		
* Of those initiated but not suppressed*:				
*Retained*, *unsuppressed viral load test reported*	*11 (6%)*	*17 (9%)*		
*Retained*, *no viral load test reported*	*14 (7%)*	*16 (9%)*		
*Transferred to another clinic*	*1 (1%)*	*6 (3%)*		
*Died*	*3 (2%)*	*0 (0%)*		
*Lost to follow-up*	*11 (6%)*	*24 (13%)*		
Initiated ≤ 90 d	136 (72%)	182 (97%)	25% (19%–33%)	1.36 (1.24–1.49)
Initiated ≤ 90 d and retained at 10 mo (secondary outcome)	121 (64%)	151 (81%)	17% (5%–23%)	1.27 (1.12–1.44)
*Of those not initiated ≤ 90 d and retained at 10 mo*:	*69 (36%)*	*36 (19%)*		
*Initiated but not retained*	*15 (8%)*	*31 (17%)*		
*Not initiated*	*54 (28%)*	*5 (3%)*		

By 90 d after study enrollment, 97% (182/187) of participants in the rapid arm and 72% (136/190) of participants in the standard arm had initiated ART, equating to a risk difference of 25% (95% CI 19%–33%) and a crude relative risk of 1.36 (1.24–1.49) (Table 2). In adjusted analysis (S1 Table), neither age, sex, nor baseline CD4 count affected these values. By 180 d, one additional patient in the rapid arm and two in the standard arm had initiated, leaving four patients in the rapid arm and 52 in the standard arm who did not initiate within the period of validity of their CD4 count results. In the rapid arm, all four were referred to a clinic nurse or doctor for clinical confirmation of TB and did not return for ART initiation. In the standard arm, 73% (38/52) of the patients who did not initiate within 180 d made no further visits to the site after the visit in which they were enrolled in the study.

**Fig 3:**
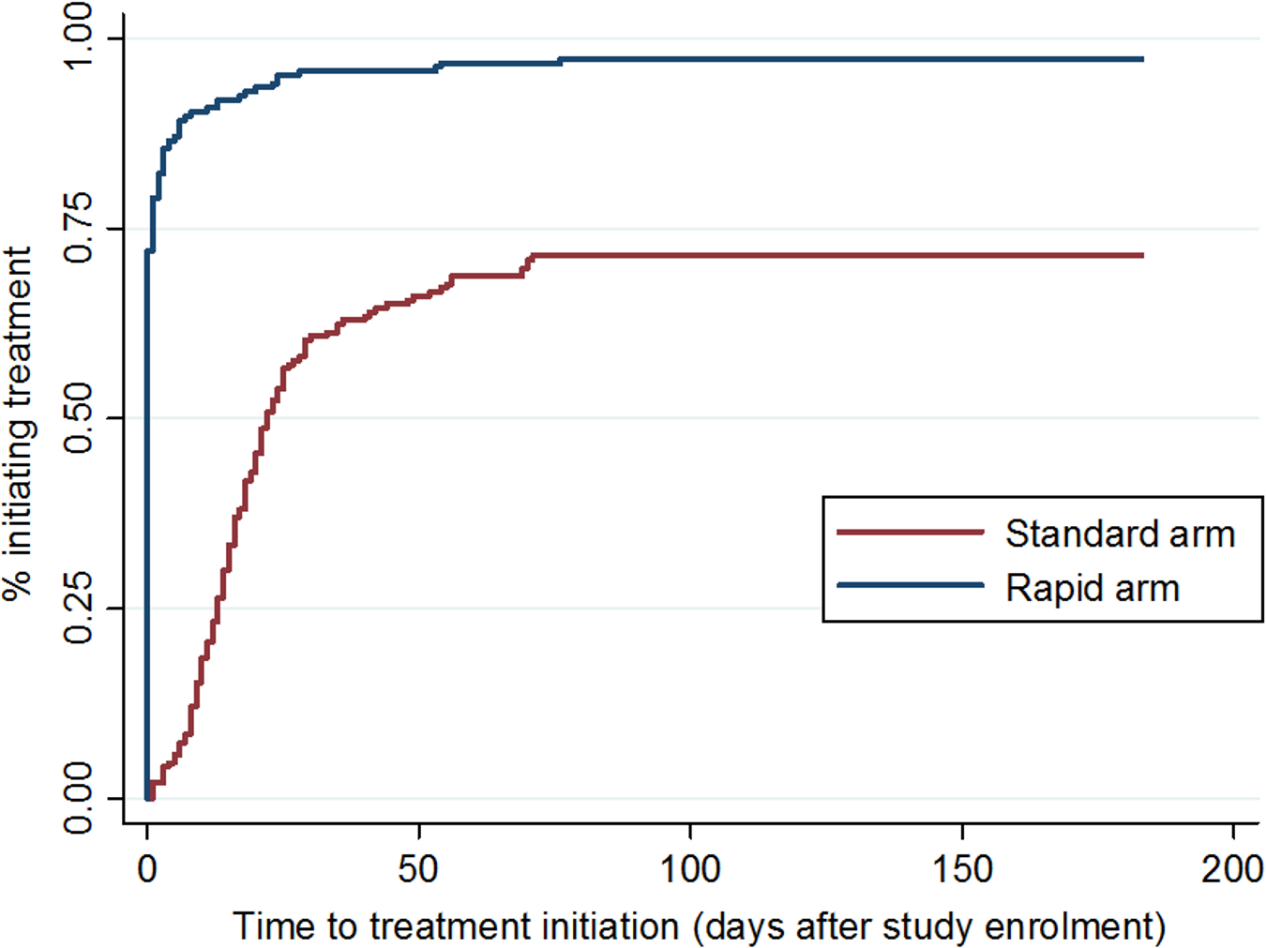
Time to ART initiation, by study arm. Cumulative incidence of ART initiation in each study arm, by number of days since study enrollment.

Fig 3 shows the cumulative incidence of treatment initiation in each study arm over the 180 d following enrollment. In the rapid arm, 72% (135/187) of patients started ART on the same day as study enrollment, an additional 7% (13/187) on the next day, and 96% (179/187) within 1 mo. In the standard arm, 58% of patients initiated within one month. The median (IQR) time to initiation in the standard arm for the subset who did initiate within 90 d (*n* = 136) was 17 (11–26) d. For rapid arm patients who did not initiate on the same day (*n* = 48), the reasons for delay were the need for clinical confirmation of TB or a Stage 3 or 4 condition or for TB treatment (25/48, 52%), insufficient time to complete all steps on the same day (6/48, 13%), patient preferences (5/48, 10%), lack of electricity in the clinic (2/48, 4%), and unknown reasons (10/48, 21%). Time to treatment initiation in the standard arm was shorter for patients who already had CD4 count results available upon study enrollment (median days 16, [IQR 11–22]) compared to those who enrolled in the study at the time of having an HIV test (22 [IQR 10–35]); the median for both types of patients in the rapid arm was 0 d (i.e., same-day initiation).

All patients in the rapid arm had the opportunity to initiate treatment on the day of study enrollment (same-day initiation) unless one of the reasons for delay listed above pertained to them. To explore whether a delay in initiation was associated with different post-initiation outcomes, we compared patients who did initiate on the same day to those who delayed for any reason. There were no differences in either the primary outcome of viral suppression or the secondary outcome of retention in care between these two groups of patients (S3 Table). Because this analysis was limited to rapid arm patients, however, it is not a randomized comparison and should be interpreted with caution.

Retention in care, defined as making a clinic visit between months 5 and 10 after study enrollment, was 81% (151/187) in the rapid arm and 64% (121/190) in the standard arm, for a risk difference of 17% (5%–23%) and a crude relative risk of 1.27 (1.12–1.44). Table 2 also indicates that 86% (31/36) of patients in the rapid arm who were not retained were lost from care after ART initiation, compared to just 22% (15/69) in the standard arm; the fall-off in the standard arm, in contrast, was mainly among those who never initiated (54/69, 78%). Although there was less loss to follow-up after initiation in the standard arm (15/190, 8% versus 31/187, 17%), this was more than offset by the higher pre-initiation loss in the standard arm (54/190, 28% versus 5/187, 3%), resulting in an overall increase in retention of 17%. Among the patients lost to care after initiation (15 in the standard arm and 31 in the rapid arm), a large majority of patients who initiated ART but were not retained in care either never came back after their initiation visit (40% of patients in the standard arm (6/15) and 45% in the rapid arm (14/31)) or came back just once (47% (7/15) and 35% (11/31), respectively), suggesting that most of these patients were never “established” on ART.

To explore further the rate of loss to care, we estimated attrition from care within the first 10 mo after initiation among the subsample of 318 patients who did initiate ART within 90 d. In the standard arm, during 1,250 mo of total person-time, 22/136 (16%) dropped out of care after ART initiation, for an attrition rate of 1.8 per 100 person-months. In the rapid arm, during 1,626 mo of total person-time, 30/182 (16%) dropped out of care, for a rate of 1.8 per 100 person-months. The hazard of attrition within the first 10 mo after ART initiation among those who initiated within 90 d did not differ between the treatment arms (HR 1.06; 95% CI 0.61–1.84). We note that this result is subject to selection bias and confounding, however, due to the exclusion of those who did not start treatment within 90 d.

In pooled analysis of both study arms, none of the variables presented in Table 1 predicted any of the outcomes reported above, with three exceptions (S2 Table). A slightly higher proportion of patients with baseline CD4 counts below 100 cells/mm^3^ initiated ART, but this difference did not persist through retention or viral suppression at 10 mo. As might be expected, patients who enrolled in the study at the time of receiving their CD4 count results (thus their second HIV-related clinic visit overall), rather than at the time of having an HIV test, were slightly more likely to achieve all three outcomes, though only for retention in care was this difference statistically significant. Finally, patients who reported being employed at the time of study enrollment, while no more likely to initiate ART, had significantly better retention in care and viral suppression than did those who reported being unemployed.

In stratified analysis (S4 Table) we observed non-significant differences in effect sizes for the primary outcome (viral suppression at 10 mo) by sex, age group, and study site. A larger effect was seen among men aged <35 y (risk difference [95% CI] 34% [12%–55%]), while little effect was seen among men or women ≥35 (5% [-9%–19%]). The effect size was also greater at the primary health clinic (21% [8%–34%]), while little effect was seen at the hospital-based HIV clinic (2% [-12%–17%]). As noted, these differences were not statistically significant, and the study was not powered to detect differences among subgroups.

In the rapid arm, for which TB diagnostic data were available, 29/187 patients (16%) presented with TB symptoms and were tested for TB using Xpert MTB/RIF. Four patients (17% of those with symptoms and 2% of all rapid arm patients) had a confirmed TB diagnosis. All four initiated ART within the 90-d outcome defined above, with a range of 11–54 d between study enrollment and ART initiation.

The results of the sensitivity analysis incorporating the six patients who were excluded after randomization for reasons other than ART eligibility or prior CD4 count, and assigning each a negative outcome, did not differ substantively from the findings presented above, with a relative risk of viral suppression by 10 mo of 1.22 [1.02–1.46].

Rapid initiation, using the procedures described above and as implemented by the study, appeared acceptable to patients at the time it was offered and feasible to implement at both study sites. We were not able to assess acceptability after patients received the intervention, as the study had no post-initiation interaction with those enrolled, and thus can surmise acceptability only on the basis of acceptance of the intervention. The study refusal rate was very low (31/603, 5%); nearly four out of five (148/187, 79%) patients offered the intervention accepted initiation on the same day or the next day, and rapid arm patients consistently expressed appreciation for the opportunity to start immediately.

All steps in the rapid initiation process were completed on the same day as study enrollment for 72% (135/187) of those in the rapid arm, demonstrating the feasibility of the intervention, at least within the context of the study. From provision of informed consent (study enrollment) to dispensing of the first supply of ARV medications, rapid initiation took a median of 2.4 (IQR 2.1–2.8) hours for those who initiated on the same day as study enrollment. This interval was shorter for patients who already had CD4 count results in hand at study enrollment (median 2.25 hours). It was longer (median 4.5 hours) for those who required a TB test and did initiate ART on the same day, but 15/20 patients requiring TB tests did not initiate on the same day. The only obstacle encountered in implementing rapid procedures was fairly frequent power outages, a common occurrence in South Africa, at Site 1, which did not have a generator for backup power supply. Most rapid instrument tests could not be performed during power outages. The rapid test instruments otherwise performed well throughout the study, and no major delays or problems arose in the acceleration of clinic procedures.

## Discussion

In this randomized controlled trial, we evaluated the effectiveness of an accelerated ART initiation algorithm that combined compressed and accelerated clinic procedures with point-of-care laboratory testing technologies that allowed eligible patients to initiate ART in a single clinic visit. This intervention increased the proportion of patients eligible for ART at study enrollment who initiated ART within 90 d by 25%, to 97% of all eligible patients and 100% of patients who were not delayed for TB treatment. By 10 mo after study enrollment, the intervention increased viral suppression among all treatment-eligible patients by 13% and retention in care by 17%. It was feasible and appeared acceptable at both study sites.

The trial demonstrated that it is possible to initiate nearly all eligible patients on ART, and to do so in a much shorter time interval than previously required. The net benefit for overall viral suppression was clinically meaningful and may underestimate the true benefits of the intervention. Both the study sites were relatively well-managed, public sector clinics, resulting in a higher rate of ART initiation in the standard arm (72%) than is found elsewhere in the country, for example in rural KwaZulu Natal Province where the rate was 59% [2]. In addition, we observed a larger effect at Site 1, the primary health clinic, than at Site 2, the hospital-based HIV clinic. Primary health clinics, which have fewer resources than hospital-based clinics but treat 85% of HIV patients in South Africa, may struggle more with loss to follow-up before treatment initiation than do hospital-based clinics, creating a greater opportunity for a service delivery intervention like RapIT to be effective. The potential for reaching younger men, who have been among the least likely to access ART under standard care [22], is another important potential benefit of rapid initiation. Additional research is needed to explore further the non-significant differences in effect that we observed in our study.

The patients who likely benefited most from RapIT were those who would not otherwise have initiated treatment at all, or who would have waited until they were sick enough to compromise their prognosis on treatment. In the standard arm, most patients who did not start treatment did not return to the study clinics for even one more visit, underscoring the importance of taking full advantage of the first visit to get as many patients started on treatment as possible. For those who would have initiated treatment, just not as soon, there is some evidence that even relatively short delays may be harmful. A recent modeling exercise using South African data estimated that compared to immediate initiation, a delay in initiating ART of 70 d would lead to a 34% increase in 12-mo mortality [22]. Delaying treatment initiation thus both deters some patients from starting at all and jeopardizes outcomes for those who do start.

We hypothesize that the delays and multiple visits patients must endure before starting ART directly deter treatment initiation. Patients who cannot afford transport fare for multiple visits, have childcare obligations at home, or risk job or wage loss if they miss too many days of work may be directly deterred from returning. Others may simply grow impatient or lose their courage or motivation, particularly if they are asymptomatic when diagnosed. These patients are likely to drift away and only return when their CD4 counts are lower and symptoms have started, or to die before treatment can be started. Our results suggest that offering the opportunity to start treatment on the spot, without delay, overcomes these barriers, without risking poorer outcomes later on.

Among patients who did initiate ART, post-initiation loss to care was higher in the rapid arm than the standard arm. This difference disappeared in the survival analysis, which controlled for number of months on ART but does not reflect the benefits of randomization. We speculate that some patients who did not want or were not ready for treatment chose to accept immediate initiation simply because it was offered or they wanted to participate in the study. For these patients, attrition from care was simply shifted from before ART initiation to after. While the intervention was successful in increasing the overall proportion of treatment-eligible patients with successful outcomes (viral suppression and/or retention in care), the rate of post-initiation attrition is a reminder that early retention in care and adherence support once patients start treatment remain high priorities for further research and intervention.

Other studies have gauged the impact on treatment uptake of a single POC technology [23] or changes in service delivery [24], but we found only one prior report of a “single-visit initiation” intervention that was similar, to some degree, to RapIT. That study enrolled pregnant women initiating ART for prevention of mother-to-child transmission in South Africa and found very high uptake of ART among women offered rapid initiation, but it did not have a comparison arm to allow an effect to be estimated [25]. A study in Tanzania and Zambia compared the effect of community support on a two-visit algorithm and reported 99% uptake of ART in both study arms [26]. Taken together, these studies imply that accelerating ART initiation is effective in a wide range of settings.

Nothing in the rapid initiation procedures used in this study differed fundamentally from existing clinic procedures. The intervention was delivered by study nurses and counselors with the same qualifications as existing clinic staff, though with study-specific training and supervision. The intervention imposed no major burdens on site management, though managerial acquiescence to the study and operational flexibility were needed to adjust the schedule and content of patient visits, staff responsibilities, and record keeping to allow for rapid initiation [27]. The main technical training required was in the use of the POC test instruments, which also required a secure location within the clinic, temperature control, and electricity.

Although South Africa has better clinic infrastructure than do many other countries in the region, the RapIT intervention does not require anything that most urban and many rural clinics cannot provide. We speculate that the RapIT intervention would be feasible and potentially even more effective in other high HIV prevalence areas, where patients travel farther to reach clinics and results from centralized laboratories take even longer to return. As the new WHO guidelines are adopted, moreover, laboratory test results may not be required prior to ART initiation for patients who are asymptomatic, reducing the need for POC technology.

The generalizability of our results is limited in several ways. The study was conducted in only two clinics in one province of one country. The trial intervention was delivered by study staff; it is uncertain if clinic staff delivering the same intervention will achieve the same outcomes (and whether their outcomes will be better or worse than those observed in the trial). As is typical in individually randomized trials of service delivery interventions, the possibility exists that quality of care in the standard arm was improved by the presence of the study, as clinic staff providing care for the standard arm may have been motivated by the study to make treatment initiation more efficient. If this occurred, the effect reported here would understate the true improvement in ART initiation that could be expected under routine implementation. As with many studies in which retention in care is an endpoint, we do not know the true outcomes of study patients who were not retained nor whether rapid arm patients who were not retained and who agreed to start treatment solely due to the presence of the study, and would otherwise not have done so, are at increased risk of developing ARV resistance. Finally, as reported above, rapid initiation under the study algorithm took 2–3 hours to complete, making same-day initiation impractical for patients who arrive late in the day (and for clinics with large numbers of such patients).

We also do not know how clinic and patient characteristics will affect the net cost and cost-effectiveness of the intervention. Most of the changes introduced in the RapIT intervention entailed only adjustments in schedules and staff time, and we speculate that these will not result in a major net change to service delivery costs. The POC instruments used in the trial require an up-front investment, but it may be possible to initiate ART in a single visit without any POC instruments if there is no CD4 count threshold for initiation, patients with TB symptoms are identified and managed separately, and ARV regimen adjustments are routinely made at the first refill visit, rather than before initiation. Costs saved by patients, who must make just one clinic visit rather than four or five, should also be taken into account.

The RapIT intervention as designed and implemented showed clinically meaningful improvements in ART uptake and viral suppression, providing “proof of principle” for a single-visit treatment initiation algorithm. Follow-on studies are needed to evaluate effectiveness and cost-effectiveness in routine practice in a variety of settings, and variations on the algorithm could also be considered. The RapIT trial has demonstrated that accelerating ART initiation can be effective and feasible in this setting and appeared acceptable to patients to whom it was offered; the next challenge will be adapting it to the range of settings and conditions found in clinics throughout Africa.

## Supporting Information

S1 Table Study outcomes adjusted for baseline CD4 count, age, and sex. (DOCX)

S2 Table Crude patient-level predictors of treatment uptake, viral suppression, and retention in care. (DOCX)

S3 Table Study outcomes stratified by immediate versus delayed initiation (rapid arm patients initiating ≤90 d only). (DOCX)

S4 Table Absolute and relative effect measure modification of primary outcome (initiated ≤90 d and suppressed by 10 mo). (DOCX)

S1 Text Research protocol. (PDF)

S2 Text CONSORT statement. (PDF)

## Abbreviations

ALT: alanine aminotransferase
aRR: adjusted risk ratio
ART: antiretroviral therapy
ARV: antiretroviral
IQR: interquartile range
CI: confidence interval
HR: hazard ratio
PHC: primary health clinic
POC: point-of-care
RapIT: Rapid Initiation of Treatment
RD: risk difference
RR: relative risk
TB: tuberculosis
