# Correction: Initiating Antiretroviral Therapy for HIV at a Patient's First Clinic Visit: The RapIT Randomized Controlled Trial

**DOI:** 10.1371/journal.pmed.1002050

**Published:** 2016-06-03

**Authors:** Sydney Rosen, Mhairi Maskew, Matthew P. Fox, Cynthia Nyoni, Constance Mongwenyana, Given Malete, Ian Sanne, Dorah Bokaba, Celeste Sauls, Julia Rohr, Lawrence Long

There are errors in the Funding section. The correct funding information is as follows: This project was supported by the President's Emergency Program For AIDS Relief and the NIH Office of AIDS Research, through NIH Grant Number U01 AI100015 to author SR at Boston University, from the National Institute of Allergy and Infectious Diseases (NIAID). Its contents are solely the responsibility of the authors and do not necessarily represent the official views of the NIAID. The funders had no role in study design, data collection and analysis, decision to publish, or preparation of the manuscript.
