# Supplementary material for: Initiating Antiretroviral Therapy for HIV at a Patient’s First Clinic Visit: The RapIT Randomized Controlled Trial
Source: PLoS Med. 2016 May 10;13(5):e1002015. doi: 10.1371/journal.pmed.1002015 (PMC4862681; doi:10.1371/journal.pmed.1002015)
Supplement: S1 Table — (DOCX) [file pmed.1002015.s001.docx]

**S1 Table. Study outcomes adjusted for baseline CD4 count, age, and sex**

| **Adjustment factor and outcome** | **Standard arm (n, %)** | **Rapid arm (n, %)** | **Crude relative risk [95% CI]** | **Adjusted relative risk [95% CI]** |
| --- | --- | --- | --- | --- |
| Baseline CD4 count |  |  |  |  |
| Initiated, retained and suppressed 10 months | 96 (51%) | 119 (64%) | 1.26 (1.05-1.50) | 1.24 (1.04-1.48) |
| Initiated within 90 days | 136 (72%) | 182 (97%) | 1.36 (1.24-1.49) | 1.35 (1.23-1.48) |
| Initiated and retained 10 months | 121 (64%) | 151 (81%) | 1.27 (1.12-1.44) | 1.26 (1.11-1.43) |
| Age |  |  |  |  |
| Initiated, retained and suppressed 10 months | 96 (51%) | 119 (64%) | 1.26 (1.05-1.50) | 1.25 (1.05-1.49) |
| Initiated within 90 days | 136 (72%) | 182 (97%) | 1.36 (1.24-1.49) | 1.19 (1.09-1.28) |
| Initiated and retained 10 months | 121 (64%) | 151 (81%) | 1.27 (1.12-1.44) | 1.26 (1.11-1.44) |
| Sex |  |  |  |  |
| Initiated, retained and suppressed 10 months | 96 (51%) | 119 (64%) | 1.26 (1.05-1.50) | 1.26 (1.05-1.50) |
| Initiated within 90 days | 136 (72%) | 182 (97%) | 1.36 (1.24-1.49) | 1.36 (1.24-1.49) |
| Initiated and retained 10 months | 121 (64%) | 151 (81%) | 1.27 (1.12-1.44) | 1.26 (1.11-1.44) |
| Baseline CD4 count, age, and sex |  |  |  |  |
| Initiated, retained and suppressed 10 months | 96 (51%) | 119 (64%) | 1.26 (1.05-1.50) | 1.24 (1.03-1.48) |
| Initiated within 90 days | 136 (72%) | 182 (97%) | 1.36 (1.24-1.49) | 1.34 (1.22-1.47) |
| Initiated and retained 10 months | 121 (64%) | 151 (81%) | 1.27 (1.12-1.44) | 1.25 (1.10-1.42) |
