# Supplementary material for: Initiating Antiretroviral Therapy for HIV at a Patient’s First Clinic Visit: The RapIT Randomized Controlled Trial
Source: PLoS Med. 2016 May 10;13(5):e1002015. doi: 10.1371/journal.pmed.1002015 (PMC4862681; doi:10.1371/journal.pmed.1002015)
Supplement: S2 Table — (DOCX) [file pmed.1002015.s002.docx]

**S2 Table. Crude patient level predictors of treatment uptake, viral suppression, and retention in care**

| **Variable** | | **N** | **N (%) achieving outcome** | **Risk difference (95% CI)** | **Unadjusted risk ratio (95% CI)** | | **p-value** |
| --- | --- | --- | --- | --- | --- | --- | --- |
| **Outcome = Initiated and suppressed viral load by 10 months after study enrolment** | | | | | | | |
| Gender | Male | 175 | 103 (58.9%) |  | 1.00 (reference) | |  |
|  | Female | 202 | 112 (55.4%) | -3.4% (-13.4%-0%) | 0.94 (0.79-1.12) | | 0.5037 |
| Age category | 35+ | 187 | 111 (59.4%) |  | 1.00 (reference) | |  |
|  | ≤35 | 190 | 104 (54.7%) | -4.6% (-14.6%-0%) | 0.92 (0.77-1.10) | | 0.3652 |
| Site | TTK | 213 | 113 (53.1%) |  | 1.00 (reference) | |  |
|  | TLC | 164 | 102 (62.2%) | 9.1% (0%-19.1%) | 1.17 (0.99-1.39) | | 0.0729 |
| CD4 category | ≤100 | 212 | 124 (58.0%) |  | 1.20 (0.99-1.44) | | 0.0582 |
|  | >100 | 162 | 91 (56.2%) | 2.3% (0%-12.4%) | 1.00 (reference) | |  |
| Reason for clinic visit | HIV test | 135 | 71 (52.6%) |  | 1.00 (reference) | |  |
|  | CD4 count | 241 | 144 (59.8%) | 7.2% (0%-17.6%) | 1.14 (0.94-1.37) | | 0.1898 |
| Housing type | Formal | 250 | 147 (58.8%) |  | 1.00 (reference) | |  |
|  | Informal | 127 | 68 (53.5%) | -5.3% (-15.9%-0%) | 0.91 (0.75-1.10) | | 0.3400 |
| Employment status | Employed | 225 | 142 (63.1%) |  | 1.00 (reference) | |  |
|  | Not employed | 152 | 73 (48.0%) | 15.1% (4.9-25.2%) | 0.76 (0.63-0.92) | | 0.0056 |
| Marital status | Married | 279 | 165 (59.1%) |  | 1.00 (reference) | |  |
|  | Not married | 98 | 50 (51.0%) | -8.1% (-19.6%-0%) | 0.86 (0.69-1.07) | | 0.1825 |
| **Outcome = Initiated ART within 90 days** | | | | | | | |
| Gender | Male | 175 | 143 (81.7%) |  | | 1.00 (reference) |  |
|  | Female | 202 | 175 (86.6%) | 4.9% (0%-12.3%) | | 1.06 (0.97-1.16) | 0.1958 |
| Age category | 35+ | 187 | 161 (86.1%) |  | | 1.00 (reference) |  |
|  | ≤35 | 190 | 157 (82.6%) | -3.5% (-10.8%-0%) | | 0.96 (0.88-1.05) | 0.3547 |
| Site | TTK | 213 | 173 (81.2%) |  | | 1.00 (reference) |  |
|  | TLC | 164 | 145 (88.4%) | 7.2% (0%-14.4%) | | 1.09 (1.00-1.19) | 0.0506 |
| CD4 count category | ≤100 | 212 | 187 (88.2%) |  | 1.10 (1.00-1.20) | | 0.0344 |
|  | >100 | 162 | 130 (80.2%) | 8% (0-15%) | 1.00 (reference) | |  |
| Reason for clinic visit | HIV test | 135 | 108 (80.0%) |  | 1.00 (reference) | |  |
|  | CD4 count | 241 | 210 (87.1%) | 7.1% (0%-15.1%) | 1.09 (0.99-1.20) | | 0.0852 |
| Housing type | Formal | 250 | 215 (86.0%) |  | 1.00 (reference) | |  |
|  | Informal | 127 | 103 (81.1%) | -4.9% (-13.0%-0%) | 0.94 (0.86-1.04) | | 0.2396 |
| Employment status | Employed | 225 | 193 (85.8%) |  | 1.00 (reference) | |  |
|  | Not employed | 152 | 125 (82.2%) | -3.5% (-11.1%-0%) | 0.96 (0.88-1.05) | | 0.3641 |
| Marital status | Married | 279 | 231 (82.8%) |  | 1.00 (reference) | |  |
|  | Not married | 98 | 87 (88.8%) | 6.0% (0%-13.6%) | 1.07 (0.98-1.17) | | 0.1221 |
| **Outcome = Initiated and retained in care at 10 months after study enrolment** | | | | | | | |
| Gender | Male | 175 | 121 (69.1%) |  | 1.00 (reference) | |  |
|  | Female | 202 | 151 (74.8%) | 5.6% (0%-14.7%) | 1.08 (0.95-1.23) | | 0.2299 |
| Age category | 35+ | 187 | 136 (72.7%) |  | 1.00 (reference) | |  |
|  | ≤35 | 190 | 136 (71.6%) | -1.1% (-10.2%-0%) | 0.98 (0.87-1.12) | | 0.8036 |
| Site | TTK | 213 | 147 (69.0%) |  | 1.00 (reference) | |  |
|  | TLC | 164 | 125 (76.2%) | 7.2% (0%-16.2%) | 1.10 (0.98-1.25) | | 0.1168 |
| CD4 count category | ≤100 | 212 | 159 (75.0%) |  | 1.08 (0.95-1.23) | | 0.2090 |
|  | >100 | 162 | 112 (69.1%) | 5.9% (0%-15%) | 1.00 (reference) | |  |
| Reason for clinic visit | HIV test | 135 | 87 (64.4%) |  | 1.00 (reference) | |  |
|  | CD4 count | 241 | 185 (76.8%) | 12.3% (2.6%-22%) | 1.19 (1.03-1.37) | | 0.0167 |
| Housing type | Formal | 250 | 187 (74.8%) |  | 1.00 (reference) | |  |
|  | Informal | 127 | 85 (66.9%) | -7.9% (-17.7%-0%) | 0.89 (0.78-1.03) | | 0.1245 |
| Employment status | Employed | 225 | 173 (76.9%) |  | 1.00 (reference) | |  |
|  | Not employed | 152 | 99 (65.1%) | 11.8% (2.4-21.1%) | 0.85 (0.74-0.97) | | 0.0173 |
| Marital status | Married | 279 | 201 (72.0%) |  | 1.00 (reference) | |  |
|  | Not married | 98 | 71 (72.4%) | 0.4% (0%-10.7%) | 1.01 (0.87-1.16) | | 0.9383 |
