# Supplementary material for: Initiating Antiretroviral Therapy for HIV at a Patient’s First Clinic Visit: The RapIT Randomized Controlled Trial
Source: PLoS Med. 2016 May 10;13(5):e1002015. doi: 10.1371/journal.pmed.1002015 (PMC4862681; doi:10.1371/journal.pmed.1002015)
Supplement: S3 Table — (DOCX) [file pmed.1002015.s003.docx]

**S3 Table. Study outcomes stratified by immediate vs. delayed initiation (rapid arm patients initiating ≤ 90 days only)**

| **Outcome stratified by adjustment factor** | **Achieved outcome**  **(n, %)** | **Crude risk difference**  **[95% CI]** | **Crude relative risk**  **[95% CI]** |
| --- | --- | --- | --- |
| Retained and suppressed 10 months |  |  |  |
| Same day initiation | 90/135 (67%) | 5% (-10-21%) | 1.09 (0.84-1.39) |
| Delayed initiation (> 1 day) | 29/47 (62%) | 1 (Reference) | 1 (Reference) |
| Retained 10 months |  |  |  |
| Same day initiation | 114/135 (84%) | 6% (-6-20%) | 1.08 (0.92-1.27) |
| Delayed initiation (> 1 day) | 37/47 (79%) | 1 (Reference) | 1 (Reference) |
