# Supplementary material for: Initiating Antiretroviral Therapy for HIV at a Patient’s First Clinic Visit: The RapIT Randomized Controlled Trial
Source: PLoS Med. 2016 May 10;13(5):e1002015. doi: 10.1371/journal.pmed.1002015 (PMC4862681; doi:10.1371/journal.pmed.1002015)
Supplement: S4 Table — (DOCX) [file pmed.1002015.s004.docx]

**S4 Table. Absolute and relative effect measure modification of primary outcome (initiated ≤ 90 days and suppressed by 10 months)**

| **Outcome** | **Standard arm**  **(n, %)**  **n=190** | **Rapid arm**  **(n, %)**  **n=187** | **Crude risk difference**  **(95% CI)** | **Crude relative risk**  **(95% CI)** |
| --- | --- | --- | --- | --- |
| <35 years | 40/92 (43%) | 64/98 (65%) | 22% (8-36%) | 1.50 (1.14-1.98) |
| ≥ 35 years | 56/98 (57%) | 55/89 (62%) | 5% (-9-19%) p=0.1835* | 1.08 (0.85-1.37) |
| Males | 43/85 (51%) | 60/90 (67%) | 16% (2-30%) | 1.32 (1.02-1.70) |
| Females | 53/105 (50%) | 59/97 (61%) | 10% (-3-24%) p=0.714* | 1.21 (0.94-1.54) |
| Male < 35 | 12/32 (38%) | 32/45 (71%) | 34% (12-55%) | 1.90 (1.17-3.08) |
| Male ≥ 35 | 31/53 (58%) | 28/45 (62%) | 4% (-16-23%) p=0.1917* | 1.06 (0.77-1.47) |
| Female < 35 | 28/60 (47%) | 32/53 (60%) | 14% (-5-32%) | 1.29 (0.91-1.83) |
| Female ≥ 35 | 25/45 (56%) | 27/44 (61%) | 6% (-15-26%) p=0.7109* | 1.10 (0.78-1.57) |
| Full service primary health clinic | 46/108 (43%) | 67/105 (64%) | 21% (8-34%) | 1.50 (1.15-1.95) |
| Hospital-based HIV clinic | 50/82 (61%) | 52/82 (63%) | 2% (-12-17%) p=0.084* | 1.04 (0.82-1.32) |
| Purpose of clinic visit = HIV test | 34/73 (47%) | 37/62 (60%) | 13% (-4-30%) | 1.28 (0.93-1.76) |
| Purpose of clinic visit = receipt of CD4 count result | 62/116 (53%) | 82/125 (66%) | 12% (0-24%)  p=0.9529* | 1.23 (0.99-1.52) |

*p value corresponds to the absolute interaction
