# Supplementary material for: Initiating Antiretroviral Therapy for HIV at a Patient’s First Clinic Visit: The RapIT Randomized Controlled Trial
Source: PLoS Med. 2016 May 10;13(5):e1002015. doi: 10.1371/journal.pmed.1002015 (PMC4862681; doi:10.1371/journal.pmed.1002015)
Supplement: S1 Text — (PDF) [file pmed.1002015.s005.pdf]

Research Protocol

Rapid Initiation of Antiretroviral Therapy to Promote Early HIV/AIDS Treatment in South Africa (RapIT Study)

University of the Witwatersrand HREC Protocol Number: M120843

BUMC IRB Protocol Number: H-31880

Federal-Wide Assurance Numbers:

FWA00000301 (Boston University Medical Campus)

FWA00000715 (University of the Witwatersrand)

November 25, 2014

Version 1.11

Table of Contents

1. Summary **4**
2. Investigators and Institutions **4**
3. Background **5**
3a. Loss to HIV treatment initiation 5
3b. Reasons for high rate of loss 6
3c. Potential solution: accelerated initiation using rapid diagnostics 7
4. Objectives **9**
4a. Primary objectives 9
4b. Secondary objectives 9
5. Study Design **9**
5a. Overview of study design 9
5b. Study site 10
5c. Study population 11
6. Procedures for Track 1 ..... **12**
6a. Overview of study procedures 12
6b. Screening, consent, and assignment to study group 14
6b i. Identification of potential study subjects (pre-screening) 14
6b ii. Screening and informed consent 14
6b iii. Questionnaire 15
6b iv. Randomization and blood draw 15
6b v. Payment 15
6b vi. Confirmation of treatment eligibility and study enrollment 16
6c. Rapid ART initiation procedures (rapid initiation group) 16
6c i. Rapid CD4 count and TB symptom screen 16
6c ii. TB test 17
6c iii. Baseline lab tests, physical exam, and educational session 18
6c iv. Decision to start ART 18
6c v. Subjects requesting or requiring a delay 18
6d. Standard ART initiation procedures (Track 1 standard initiation group) 18
6d i. Determination of treatment eligibility 19
6d ii. Remaining ART readiness and initiation steps 19
6e. Follow up 19
6e i. Direct study interaction 19
6e ii. Medical record follow up 20
7. Procedures for Track 2 ..... **21**
7a. Procedures—prospective (post) group 21
7a i. Identification of potential subjects and informed consent 21
7a ii. Questionnaire 21
7b. Procedures—retrospective (pre) group 22
7c. Follow up 22
7d. Record review
8. Data Collection and Management **22**
8a. Sources of data 22

8b. Screening form and pre-screening notebook 23
8c. Case report form 23
8e. Medical record data 24
8f. Data management 25
8f i. Study identification numbers and linking of records 25
8f ii. Data management and storage 26
8f iii. Data quality assurance 26
8f iv. Aggregate statistics 27
8f v. Destruction of data 27
9. Staff Supervision and Training 27
10. Sample Size 28
11. Outcomes and Data Analysis 29
11a. Outcomes 29
11a i. Primary outcome 29
11a ii. Secondary outcomes 30
11b. Clinical outcomes analysis 30
11c. Cost outcomes analysis 31
11d. Analysis of process indicators 31
11e. Dissemination of findings 32
12. Human Subjects Considerations 32
12a. Risks and protection against risks 32
12a i. Loss to HIV care 32
12a ii. Emotional distress 33
12a iii. Accidental disclosure of HIV status or other loss of confidentiality 33
12a iv. Time loss 34
12b. Benefits 34
12b i. Direct benefits 34
12b ii. Indirect (societal) benefits 35
12b iii. Ratio of benefits to risks 35
12c. Costs and payments to subjects 35
12d. Recruitment 35
12e. Informed consent 36
12f. Protection of confidentiality 37
12g. Data safety monitoring 37
13. References 39
14. Appendices 42

1. Summary

One of the most serious challenges facing antiretroviral therapy (ART) programs for HIV/AIDS in resource-constrained settings is the failure of ART-eligible patients to complete the steps required to initiate treatment. The high rate of loss to care of patients who are treatment-eligible at HIV diagnosis may be due in part to the large number of steps required between receiving an HIV diagnosis and obtaining the first dose of antiretrovirals (ARVs). In South Africa, these steps usually require approximately four clinic visits over a period of 2-8 weeks before a patient can start treatment. One strategy proposed for reducing losses among those eligible for ART is to simplify and condense the steps required for starting treatment. This is now possible because new, point-of-care (POC) tests for CD4 counts and TB diagnosis are available. These technologies can be combined with changes to clinic schedules to allow all steps required for ART initiation under South African guidelines (lab tests, physical exam, education) to take place on the day the patient presents for an HIV test.

This study is a randomized strategy evaluation of the feasibility, effectiveness, and cost-effectiveness of rapid ART initiation. Outpatient, non-pregnant, HIV-positive adults who come to a South African clinic for an HIV test, consent to study participation, and are eligible for ART will be randomized 1:1 to rapid ART initiation or to standard care. Those who are assigned to rapid ART initiation will have the possibility of receiving their first dose of ARVs as early as the same day, while those who are assigned to standard care will follow the clinic's usual procedures for starting ART. Rapid ART initiation for HIV-positive pregnant women, which has recently become the standard of care in South Africa, will also be assessed in a programmatic evaluation conducted alongside the randomized evaluation, with a retrospective comparison group. The primary study outcome for non-pregnant adults will be remaining alive, in care and virally suppressed 10 months after having a positive HIV test or CD4 count at the study site. The primary study outcome for pregnant women will be adherence to ART until delivery. The cost effectiveness of the rapid initiation strategy will be assessed as the cost per patient achieving the primary outcome for each population.

2. Investigators and Institutions

The study will be jointly implemented by the Boston University Center for Global Health and Development and the Health Economics and Epidemiology Research Office (HE^2RO) of the Wits Health Consortium at the University of the Witwatersrand.

<u>Principal investigator</u>:

Sydney Rosen
Research Associate Professor
Boston University Center for Global Health & Development
Boston, MA
Role on study: principal investigator

<u>Investigators (Boston University)</u>:

Matthew Fox
Associate Professor
Boston University Center for Global Health & Development
Boston, MA

Role on study: epidemiologist; statistical analyst

Investigators (University of the Witwatersrand):

Lawrence Long
Deputy Division Head
Health Economics and Epidemiology Research Office
Johannesburg, South Africa
Role on study: principal investigator for University of the Witwatersrand

Ian Sanne
Division Head
Health Economics and Epidemiology Research Office
Johannesburg, South Africa
Role on study: clinical advisor

Mhairi Maskew
Senior Researcher
Health Economics and Epidemiology Research Office
Johannesburg, South Africa
Role on study: epidemiologist; lead data analyst

3. Background

3a. Loss to HIV treatment initiation

In recent years, multiple studies have demonstrated that antiretroviral therapy (ART) for HIV/AIDS is more effective when initiated earlier in disease progression. Earlier treatment has been shown to reduce morbidity and mortality in the patient[1–4], protect the patient's uninfected partners[5], and potentially reduce overall medical care costs[6]. For treatment-eligible pregnant women who learn they have HIV during the antenatal period, earlier initiation of ART significantly reduces the risks of mother-to-child-transmission[7].

For HIV care and treatment providers in high-prevalence settings, this research has underscored the urgent need to identify strategies that will achieve earlier treatment initiation among those already diagnosed. A recently published review of the literature on pre-ART retention in care found that roughly one third of adult patients in sub-Saharan Africa who had already been determined eligible for ART did not start ART within the studies' follow-up periods[8]. If those diagnosed with HIV but not assessed for treatment eligibility had been added, this proportion would have been far higher. The result is persistently late initiation of treatment, even for those already accessing healthcare.

This problem is particularly apparent in South Africa, the country with the largest burden of HIV in the world[9]. Despite aggressive HIV testing campaigns and the availability of ART in most districts, median starting CD4 counts remain well below treatment eligibility thresholds[10,11]. Nationally, the average median starting CD4 count in studies published between 2007 and 2009 was 95 cells/mm^3 (IQR 72-114)[12]. In 7 studies from South Africa included in the review of pre-ART retention in care mentioned above, a median of 32% (range 16-61%) of patients who were ART-eligible under the more stringent eligibility guidelines previously in place in South Africa (CD4 ≤ 200 for all adults) did not initiate

treatment within the period of study observation (3-12 months after confirmation of eligibility)[8]. In studies we conducted at a large public treatment facility in Johannesburg, we found that:

- More than half (54%) of those who seek HIV testing at the outpatient clinic are already treatment-eligible[13];
- Nearly half of those who are eligible for ART (49%) do not return to obtain their CD4 count results within 3 months[13], and are thus not aware of their need for treatment; and
- Even among those who do receive their CD4 count results and are eligible or fast-tracked for ART (CD4 $\leq$ 250) fewer than half (46%) initiate ART within four months of HIV testing[14].

This problem is likely to be even worse among patients who obtain an HIV test from a mobile or stand-alone HIV counseling and testing site, and thus must be referred elsewhere for a CD4 count and other services. South Africa's recent raising of the treatment eligibility threshold to CD4 ≤ 350 is likely to exacerbate this problem further, as more asymptomatic patients become eligible.

Failure to initiate ART promptly is also an acute problem amongst pregnant women who are diagnosed with HIV during antenatal care. A study in Cape Town, South Africa found that only half (51%) of pregnant women who were eligible for full HAART (three drug treatment regimen rather than PMTCT regimen) under previous guidelines (CD4 count<200 cells/mm^3) initiated treatment prior to delivery[15], increasing the risk both to themselves and to their infants.

3b. Reasons for high rate of loss

Although studies in different parts of Africa have identified several psycho-social and structural reasons for failing to start ART[16], one major barrier is clearly the daunting number of steps required between receiving an HIV diagnosis and obtaining the first dose of ARVs. In South Africa, these steps include:

1) Confirmation of treatment eligibility (CD4 count);
2) TB diagnosis and treatment initiation if indicated;
3) Hemoglobin, liver function, pregnancy, and creatinine clearance tests;
4) General medical exam; and
5) Several (typically 3-4) wellness/treatment readiness/adherence sessions[17].

Except for the sickest patients, these activities usually require several clinic visits and a 1-2 month interval to complete even if patients adhere to the clinic's schedule. Standard laboratory technologies and procedures in use throughout sub-Saharan Africa typically require patients to make two clinic visits per diagnostic test, one to provide a sample and another to receive results. As laboratories have struggled to keep pace with the expansion of treatment programs, the time interval between these two visits has increased in many locations. In the research team's experience in South Africa, completion of a baseline CD4 count for determination of treatment eligibility can take between 2 days and 4 weeks, depending on the location of the lab, the time required to process the sample, and the promptness of the patient's return. Similarly, diagnosis of pulmonary TB requires anywhere from 2 days to 6 weeks or longer, depending on the type of TB (smear positive or smear negative), backlog at the laboratory, and delays in communicating results to the treatment site and thence the patient. Since up to 70% of HIV patients may be considered TB suspects and require TB diagnosis even though <20% have TB[18], TB diagnosis also causes substantial delays in ART initiation and leads to high loss to follow up from both HIV and TB treatment.

At the treatment site in Johannesburg where the study team is based, the average interval between the first CD4 count that indicates treatment eligibility and treatment initiation is 42 days among those who do start treatment within 16 weeks[14] and entails an average of 4 visits to the site. Even very sick patients usually face a delay of at least 2 weeks before initiation. In addition to delay in starting treatment, each clinic visit entails time, transport costs, and possibly child care and other costs for the patient[19]. Previous research at the same site estimated that the median cost to an ART patient per clinic visit was R29 ($4.15) (inflated to 2011 prices) for transportation alone[20], the equivalent of roughly a third of a day's wage for a domestic worker. Each visit also required a median of two hours' travel time. A more recent study found that, due largely to waiting time, the average duration of a routine clinic visit for a stable ART patient at this site is just under 6 hours; even simply picking up medication, without any clinical consultation, requires nearly 3 hours[21]. Requiring multiple visits before dispensing medications thus constitutes a major obstacle to treatment initiation, in addition to other possible reasons for loss to follow up.

3c. Potential solution: accelerated initiation using rapid diagnostics

One strategy proposed for reducing loss to ART initiation among those eligible is to shorten the time interval and simplify the process required for initiation, ideally allowing ARVs to be dispensed at the same visit as the HIV test or, if the patient was previously diagnosed, first visit for HIV-related care. Acceleration of the required steps is now possible under existing South African treatment guidelines due to the advent of new, rapid diagnostic tests. All the laboratory tests required for treatment initiation can now be done quickly, using the following equipment, all of which has been approved for use in South Africa:

- Assessment of CD4 count to determine ART eligibility with the Pima™ Analyzer (Alere)[22–26] (http://pimatest.com/en/pima-platform/pima-analyser.html). The Pima assay uses a capillary bleed or venous blood sample and takes 20 minutes for results to be available once the cartridge containing sample has been inserted into the instrument.
- TB diagnosis with Xpert© MTB/RIF (Cepheid) [18,27–32] (http://www.cepheid.com/media/files/eu/brochures/XpertMTB_Broch_R9_EU.pdf) Xpert uses a sputum sample and takes approximately 1 hour and 45 minutes to complete[28] once the cartridge containing the sample has been inserted into the instrument and 2.5 hours overall, including sample collection and delivery of results to the patient[33]. The World Health Organization has endorsed the use of Xpert as the first-line TB diagnostic test in HIV-positive TB suspects[34], and South Africa has announced plans to utilize Xpert at National Health Laboratory Service laboratories nationally[35].
- Liver function (alanine aminotransferase) and creatinine clearance using Reflotron Plus (Roche) [26,36] (http://www.roche-diagnostics.co.in/Products/Pages/ReflotronPlusDry.aspx).
- Hemoglobin using the HemoCue® Hemoglobin System[26] (http://www.hemocue.com/international/Products/Hemoglobin-1155.html).

These tests, combined with those already routinely used as point-of-care rapid tests (e.g. pregnancy), should allow full laboratory evaluation for treatment eligibility and treatment regimen selection to be completed within roughly a half-day interval. The non-laboratory steps in the initiation process can also be completed rapidly if service delivery schedules are deliberately arranged to do so. In a facility with a rapid-test equipped laboratory and willingness to manage schedules accordingly, it is thus now possible to initiate ART on the same day as the HIV test for patients who are treatment-eligible at HIV diagnosis.

Several recent studies provide some initial evidence that the use of rapid diagnostics may accelerate treatment initiation in South Africa. Figure 1 summarizes the results of four evaluations of the impact of rapid CD4 counts on uptake of care.

Figure 1. Impact of rapid CD4 counts on uptake of care in South Africa

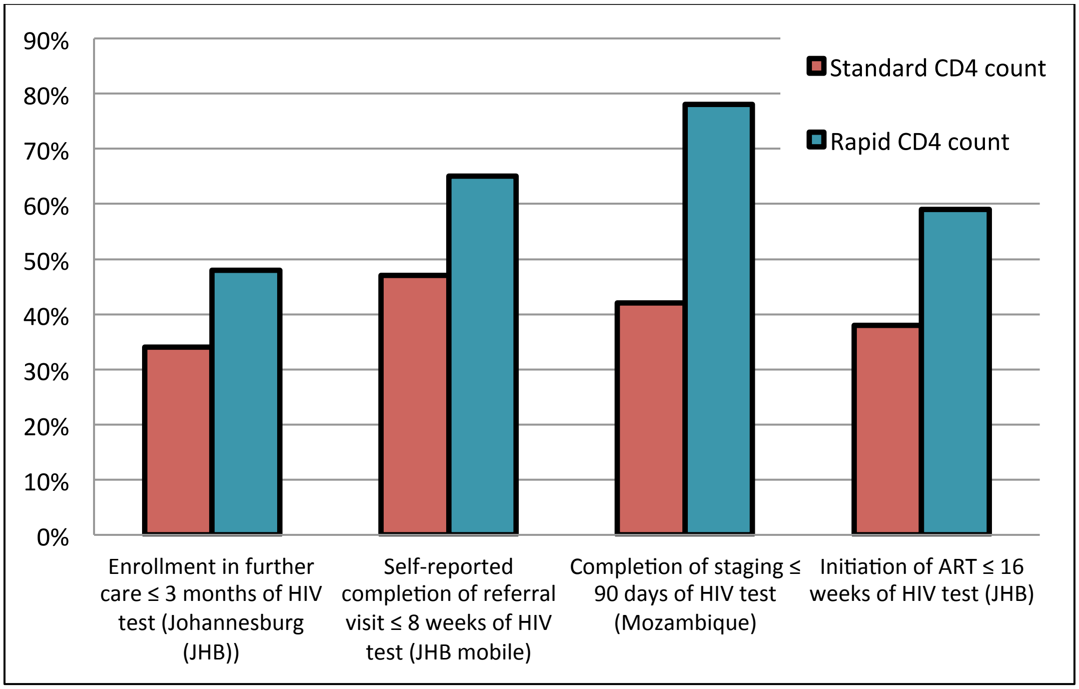

Sources: Faal et al [37]; Larson et al [38]; Jani et al [24]; Larson et al [14]

An evaluation of a pilot program allowing same-day treatment initiation for eligible pregnant women in Cape Town, which incorporated Pima™ CD4 counts but did not use rapid tests for TB or other indicators, found that of 101 treatment-eligible pregnant women, 89 (88%) did initiate ART on the same day as their HIV test[39]. Although less comprehensive than what is proposed here and observational in design, these preliminary studies suggest that rapid diagnostics are acceptable to patients and facilitate uptake of treatment.

Same-day treatment initiation could increase treatment uptake, improve patient outcomes, and save resources for both the patient and provider. It also raises a number of implementation questions, however, including:

- Will patients accept rapid treatment initiation?
- Will the availability of rapid initiation reduce losses to care prior to starting ART?
- Will rapid initiation improve overall treatment outcomes, or will an increase in early attrition after starting ART offset the gains in the pre-ART period?
- Will the feasibility and uptake of rapid initiation be the same for pregnant women as for non-pregnant adults?
- Will rapid initiation be affordable and cost-effective compared to standard care, taking into account the costs of all medical care provided before and after starting treatment?

Some patients who are offered rapid ART will likely agree to start treatment immediately even if for social, psychological, economic, or other reasons they are not personally ready. Under standard care these patients might not have started treatment at all, or started later. Under rapid initiation, they may instead accept the first month or two of medications but then drop out. It is therefore important to evaluate not only the immediate acceptability and feasibility of rapid ART initiation, but also whether it can generate equally good medical outcomes, as indicated by viral suppression, which is the basic goal of antiretroviral therapy.

4. Objectives

4a. Primary objectives

The primary objectives of this study are to:

1. For non-pregnant adults, compare the proportion of ART-eligible patients who are alive, in care, and virally suppressed within 10 months of having an HIV test or enrolling in HIV care between ART-eligible patients offered rapid ART initiation or standard ART initiation
2. For pregnant women, estimate the proportion of HIV-positive patients who adhere to ART until delivery, as indicated by monthly medication pickup.

4b. Secondary objectives

1. For non-pregnant adults, compare the cost per patient alive, in care, and virally suppressed within 10 months of an HIV test or enrollment in HIV care for those offered rapid ART initiation to the cost for those offered standard care.
2. For pregnant women, estimate the cost per patient adhering to ART through delivery and the cost per infant infection avoided, using estimated vertical transmission rates.
3. Estimate the proportion of patients alive, on ART, and virally suppressed at 6 month intervals after treatment initiation and at the final date of data censoring.
4. Estimate the average time interval between HIV test or first HIV or ANC care visit and treatment or PMTCT initiation before and after rapid ART initiation became the guideline procedure for pregnant women.
5. If enrollment numbers allow, among pregnant women, stratify the primary outcome (the proportions of subjects achieving retention on ART or PMTCT regimen until delivery) by CD4 count > 350 and ≤ 350.
6. Compare the average gestational age at ART initiation and average number of weeks on ART prior to delivery for pregnant women before and after rapid ART initiation became available.
7. Identify patient-level predictors of treatment uptake, retention in care, and viral suppression.
8. Describe the prevalence of TB symptoms and confirmed TB, time to initiation of TB treatment, and time to initiation of ART among patients with TB.
9. Evaluate the feasibility of the rapid initiation strategy in terms of patient acceptance, actual time between HIV test/enrollment in care and dispensing of first dose of ARVs, and patient costs incurred.

5. Study Design

5a. Overview of study design

The study will be divided into two tracks, based on the study populations to be included.

Track 1: *Non-pregnant adults*. Track 1 will be a randomized comparison of rapid initiation of ART to the current standard of care for non-pregnant, treatment eligible adults. Patients testing positive, providing a blood sample for a CD4 count, or receiving a CD4 count result will be individually randomized 1:1 to either the rapid ART initiation strategy (rapid group) or the standard ART initiation strategy (standard group). Patients in the rapid group who are found to be eligible for ART will be offered the opportunity to initiate ART in the fastest time feasible given individual circumstances, and whenever possible on the same day as study enrollment. Patients in the standard group will be offered standard treatment initiation as currently implemented by the study site.

Track 2: *Pregnant women*. Track 2 will be a programmatic evaluation of rapid initiation of ART for pregnant women. Immediate ART initiation on the day of a woman's first antenatal care visit was introduced in South Africa in a guideline revision in April 2013 and is now the standard of care at the study site. Guidelines call for women to remain on ART for the duration of pregnancy regardless of their initial CD4 count. Adherence to ART during pregnancy, as measured by monthly medication pickups, will be compared to adherence before rapid initiation was offered.

In Track 1, those in the rapid initiation group will be offered a rapid CD4 count, rapid TB test if needed, and other rapid tests and same-day physical exam, counseling, and adherence education on the same day as study enrollment, allowing immediate ART initiation if eligible. Those in the standard group will follow the standard-of-care visit schedule and procedures, which also include a CD4 count, TB test if needed, other tests, physical exam, counseling, and adherence education, though not on a rapid schedule. Subjects in Track 1 will be prospectively followed for a minimum of 10 months. Follow-up (after treatment initiation) will be passive and by medical record review only, to prevent biasing outcomes through active follow-up and maximize the extent to which study results reflect what could be expected in a routine implementation setting. An additional retrospective comparison group for Track 1 will be enrolled comprised of patients who became eligible for ART at one of the two study sites prior to the launch of the study.

Track 2 will be an observational evaluation only. Data from before the guideline change will be collected retrospectively, from existing registers and records at the site. Data from after the guideline change will be collected prospectively from questionnaires and newly-accrued medical record data. The study will not provide any direct clinical interventions for Track 2.

Unit cost data will be collected for all resources used for patient care for both study tracks, including all drugs, diagnostics, clinical staff time, and infrastructure and other fixed costs. Quantities of resources used will be collected from study case report forms at the end of the follow up period. An average cost per patient and cost per outcome will then be calculated and compared between the strategies.

5b. Study site

The study will be conducted at:

1. Thuthukani Primary Health Clinic, Ivory Park, South Africa**.** Thuthukani is public-sector primary care clinic and HIV comprehensive care management and treatment (CCMT) site under the authority of the

City of Johannesburg and the Gauteng Province Department of Health. It is located in a densely-populated, under-served community in the area between Johannesburg and Pretoria known as Midrand. Thuthukani maintains a large antenatal care clinic with prevention of mother-to-child transmission (PMTCT) services and provides TB treatment and other primary care.

By July 2012, Thuthukani had started almost 7000 adult patients on ART. It currently initiates roughly 840 non-pregnant adult patients and 120 pregnant women onto ART annually. Thuthukani was selected as an appropriate site for this study due to the availability of general outpatient HIV testing, HIV testing for pregnant women, TB treatment, and ART in a single facility, allowing rapid initiation for both study populations at a single site. It is a primary health clinic located in a densely-populated, under-served, township setting typical of urban sub-Saharan Africa, and study results should thus be generalizable to other areas of South Africa and the region.

2. Themba Lethu Clinic, Helen Joseph Hospital, Johannesburg. Themba Lethu Clinic (TLC) is a public sector HIV comprehensive care management and treatment (CCMT) site located in an academic hospital under the authority of the Gauteng Province Department of Health. It serves an urban population in Johannesburg and is one of the largest HIV clinics in the country, initiating nearly 10,000 adult patients onto ART annually. It was selected as a site for Track 1 of this study due to its large patient volume, existing point-of-care laboratory infrastructure, and difference in facility characteristics from the other study site (Thuthukani). It is typical of a large hospital-based HIV clinic in South Africa.

Thuthukani Primary Health Clinic and Themba Lethu Clinic both fall under the FWA of the University of the Witwatersrand Human Ethics Review Committee (Medical). A letter confirming the local government authority's agreement for each site to participate in this study will be submitted to the BUMC IRB and Wits HREC prior to study enrollment at that site.

5c. Study population

The study population will be adults who test positive for HIV at the study site's walk-in HIV voluntary counseling and testing (VCT) service and adults who have previously tested positive for HIV but are providing a blood sample for a CD4 count or receiving CD4 count test results. Non-pregnant patients will be eligible for enrollment in Track 1 and pregnant patients will be eligible for enrollment in Track 2.

Inclusion criteria:
- Adult patients ($\geq$18 years)
- For Track 1, tested HIV-positive at study site's outpatient testing service on day of study enrollment or previously tested HIV-positive but providing a blood sample for a CD4 count or receiving CD4 count test result on day of study enrollment
- Eligible for antiretroviral therapy under prevailing South African guidelines*

Exclusion criteria:
- Currently on ART or on ART at any time in the past 12 months (three-drug combination; previous PMTCT regimen exposure for an earlier pregnancy is not an exclusion criterion)
- Stated intention to seek further HIV care during the next 12 months or antenatal care before completion of the current pregnancy at another site, not at the study site
- Not physically or emotionally able to participate in the study, in the opinion of the investigators
- Not willing or able to provide written informed consent to participate in the study

- Previously enrolled in the same study
- Diagnosed with drug-resistant TB or on MDR-TB treatment
- For Track 1 only, pregnant (pregnancy is an exclusion criterion for Track 1 because treatment guidelines for pregnant women differ from those for non-pregnant adults)

**Current South African treatment eligibility criteria for non pregnant HIV-positive adults are:*

- *CD4 count ≤ 350; or*
- *Tuberculosis, regardless of CD4 count; or*
- *WHO Stage 4 or Stage 3 condition, regardless of CD count (hereafter "Stage 4/3")*

All pregnant HIV-positive women are eligible for ART.

Subjects in Track 1 will be followed prospectively and will be asked for written informed consent prior to study enrollment. An additional Track 1 retrospective comparison group will be enrolled comprised of patients who became eligible for ART at one of the two study sites (Thuthukani Clinic) prior to the launch of the study, with data collection limited to retrospective medical record review. In Track 2, subjects offered rapid initiation by the study clinic under the new standard of care will be followed prospectively and asked for written informed consent before study enrollment. For subjects in the comparison group in Track 2, data will be collected retrospectively through medical record review only. The study team will have no contact with these subjects, and informed consent will not be sought.

6. Procedures for Track 1

6a. Overview of study procedures

Figure 2 illustrates the main steps in the study for the prospective groups in Track 1 (non-pregnant adult population). Each step is described in detail in the following paragraphs. Track 2, which is an observational pre/post evaluation, is also described below.

Figure 2. Procedures for Track 1 (Non-Pregnant Adults)

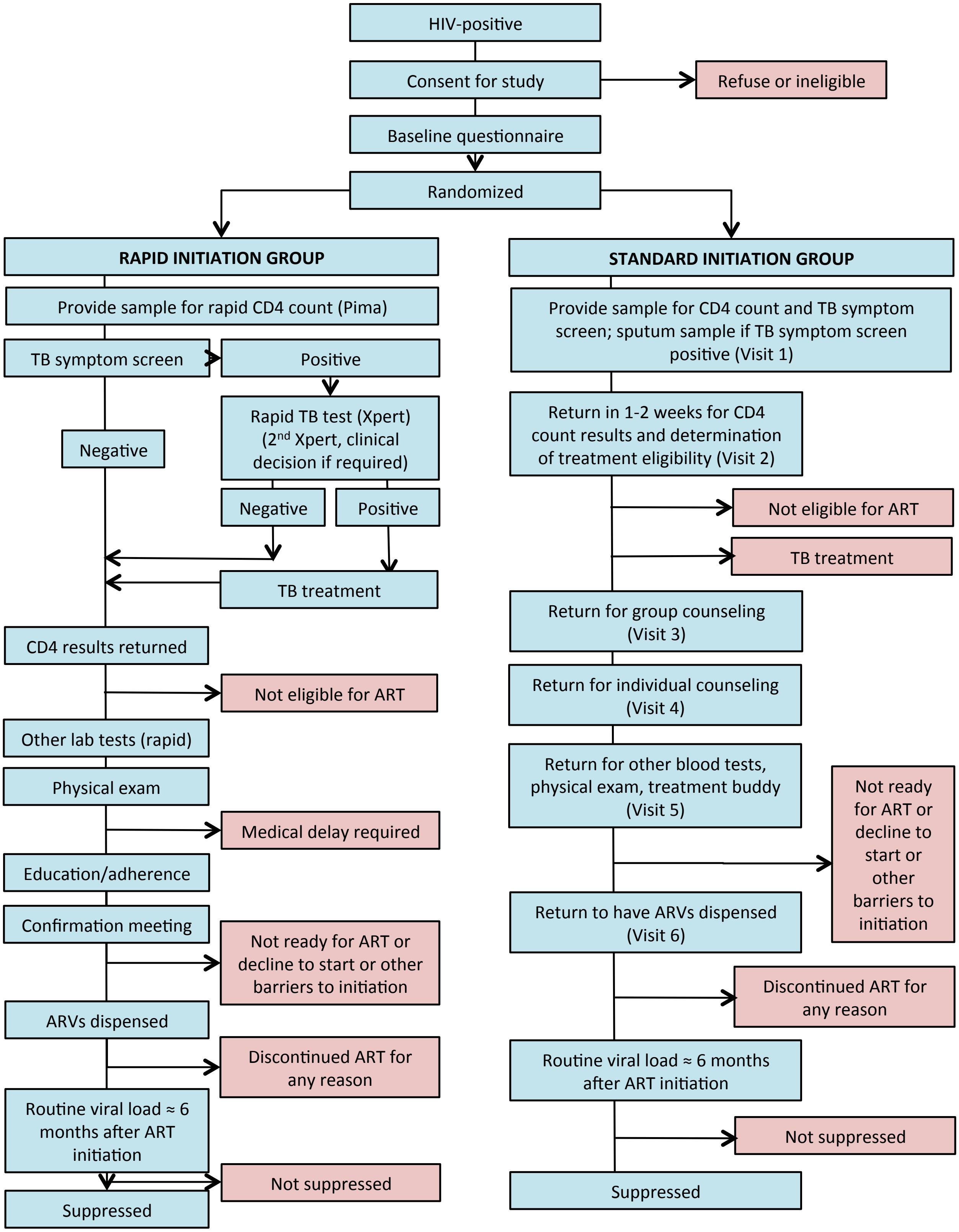

6b. Screening, consent, and assignment to study group

Procedures for subjects enrolled in Track 1 are described below.

6b i. Identification of potential study subjects (pre-screening)

Track 1 will recruit non-pregnant patients who, on the day of study screening:

- Have a positive HIV test at the VCT service at the study site; or
- Have blood drawn for a CD4 count; or
- Have returned to the study site for a CD4 count result and it is ≤ 350.

Patients are not eligible for study screening if they have returned to the site to receive a CD4 count result on the day of study screening and the CD4 count is > 350.

At the study site, the site's staff will inform potentially eligible patients about the study immediately after routine post-test counseling, CD4 count blood draw, or return of CD4 count results. The site staff will explain that a study is underway and that patients who voluntarily enroll in the study and are found to be eligible for ART may have the opportunity to initiate treatment immediately, rather than following the standard schedule of visits. Patients who indicate that they may be interested in participating in the study will be referred to a study assistant. Those who do not wish to participate will proceed with a standard pre-ART care visit.

Site counselors who perform the pre-screening will record in a notebook (pre-screening log) patients whom they do not refer for screening, to allow the study to collect aggregate statistics about the overall patient population and compare it to the screened population. Fields that will be recorded in this notebook are date, sex, age in years, and primary reason for not being screened. (We anticipate three main reasons: patient's emotional condition; patient's physical condition; refusal. An "other" field will allow the counselor to record any other reason for not referring the patient to the study assistant for screening.) No identifiers of any kind will be collected in this notebook, which is intended to provide only aggregate information.

6b ii. Screening and informed consent

Upon referral to the study assistant, patients will receive a more complete description of the study, including the details of why it is being done, the process of randomization, the steps that will occur for patients in each group, and the need for written informed consent. They will be assured that participation is voluntary and that they can withdraw from the study at any time, without affecting the quality of care provided by the site. They will also be offered the opportunity to ask questions. If the study assistant concludes that all other inclusion/exclusion criteria have been met (with the exception of treatment eligibility) they will then be asked to provide written informed consent to participate. The study assistant will complete a screening form to record study eligibility for each patient screened. The screening form will not collect any identifiable information pertaining to individual patients prior to receipt of written informed consent.

Patients will be recruited consecutively, as they arrive at the study site. Due to the volume limitations of the technology to be used in the study, however, recruitment may be staggered over the course of each day to ensure that patients randomized into the rapid initiation group can actually be offered same-day

initiation. Patients who arrive late in the day and are randomized to rapid initiation (Track 1) will be asked to return the next day, or as soon as possible, to complete all procedures.

The study information sheet and consent form will be translated into the South African languages most commonly spoken by patients at the study site. These are Isizulu, Xitsonga, and Sesotho, but additional or other languages will be included if necessary. Translated consent documents and attestation of consent accuracy will be submitted to the BUMC IRB and Wits HREC prior to use with any study subjects.

For patients who decline to participate in the study (consent refused), the study assistant will indicate the refusal on the screening form.

6b iii. Questionnaire

After a subject consents to be in the study, the study assistant will administer a short questionnaire documenting the subject's basic demographic and socioeconomic characteristics, primary reason for seeking an HIV test or HIV care, and costs incurred per clinic visit. Height and weight will be recorded to help assess balance between the two study groups (rapid and standard). As the questionnaire elicits only simple responses and will be completed by the study assistant not the subject, we do not intend to translate the questionnaire itself into subjects' first languages. Instead, for subjects who do not speak English, the assistant will ask the questions in one of the commonly-understood languages used by the study population, and then record the answers in English on the questionnaire. This approach has been used successfully in other studies in similar populations (e.g. BUMC H-24379/HREC M041116).

(Note: the questionnaire is included in the study case report form in the appendix to this protocol.)

6b iv. Randomization and blood draw

After the questionnaire has been completed, subjects will be randomized to the rapid or standard groups of the study. Randomization envelopes will be generated in blocks of 6 and kept at the study site. For each enrolled subject, the study assistant will open the next envelope in sequential order, read the randomization group, and record it on the case report form.

Depending on the randomization group, the study assistant will then ask subjects in the standard group to return to the clinic nurse for a blood draw for a standard CD4 count and subjects in the rapid group to go to the study nurse for a blood draw for a rapid CD4 count. Following the blood draw, the study assistant or study nurse will inform subjects of their group assignment. Standard group subjects will be asked to proceed with a standard pre-ART care visit. The study assistant will repeat the explanation of next steps in the study, which are limited to review of medical records by the study team, and thank them for their willingness to participate in the study. The study team will have no further personal contact with patients in the standard group.

6b v. Payment

Following the blood draw, reimbursement to consented study subjects of R100 (approximately $12.50) will be provided in the form of a voucher to a nearby supermarket or cell phone air time. An information card will be provided with the voucher, explaining where it can be used and noting that even though the voucher is identical to those sold as gift cards for all purposes, if it is used at the store

that is closest to the study clinic, the person redeeming it may be assumed to be participating in an HIV-related study.

6b vi. Confirmation of treatment eligibility and study enrollment

Under South African guidelines, ART eligibility is based primarily on CD4 count. Since a rapid CD4 count is part of the intervention for patients in the rapid group but not for those in the standard group, consent will take place before treatment eligibility is known. All subjects who meet other inclusion/exclusion criteria will be asked for consent, but only those who are treatment eligible will be enrolled in the study.

Procedures for performing a CD4 count are described below. Subjects in the rapid group will be informed as soon as their CD4 count result is available if they are eligible for treatment. Those who are not eligible on the basis of CD4 count and do not have symptoms of TB or a WHO Stage 4/3 condition will not be enrolled in the study. They will be given their CD4 count results following the clinic's standard procedures for returning laboratory results and asked to proceed with a standard pre-ART care or antenatal care visit.

Subjects in the rapid group who are eligible for treatment on the basis of CD4 count will be enrolled in the study and continue with study procedures as described below. Subjects in the rapid group who are not eligible for treatment on the basis of CD4 count but have symptoms of TB will be asked to remain in the clinic until initial TB test results are available (approximately 2 hours). Subjects who report symptoms that may indicate a Stage 4/3 condition will be examined to determine if a condition is present. Those diagnosed with TB or a Stage 4/3 condition will be enrolled in the study, while those without will be asked to proceed with a standard pre-ART care visit.

Subjects in the standard group of Track 1 will be advised to follow the standard schedule of clinic visits, which is to return to the site for their CD4 count results in 1 week. The study team will monitor CD4 count and TB test results and Stage 4/3 condition diagnoses and will enroll in the study only those who are eligible for treatment.

6c. Rapid ART initiation procedures (rapid initiation group)

The left side of Figure 1 illustrates rapid initiation procedures for the rapid group in Track 1.

6c i. Rapid CD4 count and TB symptom screen

The first step for rapid initiation will be a venous blood draw for a rapid CD4 count (and for other treatment initiation tests for those found eligible for treatment). For those offered rapid initiation, the blood sample will be processed immediately, on site, using the Pima Analyzer, rather than being stored for later transport to an off-site laboratory. The processing time for the Pima test is 20 minutes. In a validation study of Pima used at point of care clinics in South Africa, 7-9% of tests could not be read or had invalid results, due primarily to user errors in obtaining blood samples or filling cartridges[40]. Careful training will be provided to study staff to minimize this type of error. A second Pima test will be run for subjects for whom the first Pima result is invalid. In addition, a blood sample will be sent to the off-site National Health Laboratory Service (NHLS) laboratory for all subjects in the rapid group for a confirmatory CD4 count. NHLS lab results will be monitored by the study team, and any subject found

not eligible for treatment using Pima but eligible based on lab results will be contacted by the study assistant and asked to return to the clinic to initiate treatment following rapid initiation procedures.

Patients who already have with them a valid CD4 count result (sample taken within the previous 6 months) will not have an additional CD4 count, though a blood sample will still be drawn for other required laboratory tests.

While awaiting CD4 test results, the study nurse will administer a TB symptom screen following South African guidelines for the definition of symptoms, for all subjects not already on TB treatment. Subjects with any TB symptoms will be asked to provide a sputum sample. The study nurse will also ask the subject about symptoms that might indicate a Stage 4/3 condition. Subjects who report such symptoms will be given a physical examination to determine if the condition exists and what treatment is needed.

Following the TB symptom screen and sputum collection and self-report of symptoms, patients will be asked to wait for a few minutes until their CD4 count is available, if it is not yet ready. Patients who have a CD4 count that makes them ineligible for treatment and do not have symptoms of TB or a Stage 4/3 condition will be informed of their CD4 count, thanked for their willingness to participate in the study, and asked to proceed with a standard pre-ART care visit. Those for whom a sputum sample has been sent for testing with Xpert will be asked to remain at the clinic until Xpert results are ready.

Patients eligible for treatment (CD4≤350 or TB or Stage 4/3 condition diagnosis) will also be informed of their CD4 count and asked to continue to the next step of the study.

6c ii. TB test

Sputum samples for patients with TB symptoms will immediately be sent to the on-site laboratory for testing using Xpert MTB/RIF. Each patient will be asked for two sputum samples to allow two Xpert tests to be run[32]. Those with at least one positive Xpert test will be considered to have active TB. Those with two negative tests will be considered not to have TB. Those with inconclusive test results will be diagnosed clinically by the study nurse or the site doctor. Patients diagnosed with TB will be told of their results and escorted by the study assistant to the site's TB clinic, where TB treatment can be initiated.

A third sputum sample for all patients diagnosed with TB will be sent to the off-site National Health Laboratory Service laboratory for smear microscopy and culture if required. The NHLS lab results will be monitored by the study team. Any positive TB diagnosis not already made by the study nurse will be conveyed immediately to the site, and the patient will be contacted by the study assistant and asked to return to the clinic to initiate TB treatment.

The study site's standard procedures will be followed with regard to TB treatment. Under current South African guidelines, two weeks (14 days) of TB treatment is required prior to initiating ART for patients with a CD4 count ≤ 350 and eight weeks of TB treatment is required for patients with a CD4 count > 350. Study subjects diagnosed with TB and with CD4 counts ≤ 350 will be asked to return to the clinic between 14 and 28 days after study enrollment to continue with rapid ART initiation. Those returning in more than 28 days will be offered standard ART initiation. Subjects with TB and CD4 counts > 350 will be advised to return for TB treatment only, then continue with standard ART initiation. Patients already on TB treatment but for less than 14 days at the time of study enrollment will also be asked to return once they have reached 14 days of TB treatment.

6c iii. Baseline lab tests, physical exam, and educational session

Subjects who are eligible for ART will be enrolled in the study once treatment eligibility is confirmed. Using the same blood sample drawn earlier, the study nurse will perform the required baseline blood tests (creatinine, hemoglobin, ALT) and will perform a pregnancy test for female subjects in Track 1. (Track 1 rapid group subjects who are found to be pregnant will be transferred to Track 2 at this time.)

Subjects will then be examined by the study nurse, who will conduct the standard ART readiness physical examination. Either before or after the medical exam, depending on each day's patient flow, each subject will also meet individually or in a small group with the trained study assistant for an educational and counseling session on antiretroviral therapy, the importance of adherence and disclosure, and other actions to promote health while on ART. This will be a concise version of the education and counseling typically provided over the course of three visits under standard care. During this session, each subject will have an opportunity to ask questions and discuss concerns with the study assistant.

6c iv. Decision to start ART

After the baseline lab tests, physical exam, and education and counseling session, subjects will be asked to wait briefly while the study nurse reviews the laboratory and examination results and determines whether treatment can be initiated. She will then meet individually with the subject to convey the results of the lab tests and confirm that the subject still wishes to initiate ART immediately. Subjects who choose not to start ART immediately will be managed as described under "subjects requesting a delay" below.

Finally, the study nurse will write a prescription for the first month's ARVs, following South African guidelines for allowable first-line regimens. This prescription can be filled immediately at the study site's pharmacy, allowing the first dose to be taken on the same day.

6c v. Subjects requesting or requiring a delay

The rapid treatment initiation process is designed to occur in one day, over a period of approximately 4-5 hours. Some patients may require more than one day, however, for example if they arrive for an HIV test late in the afternoon or cannot take several hours away from work or child care on that day but can do so later in the week. Others may need time to disclose to or obtain support from friends or family or to consider their decision. In addition, subjects with TB must have 14 days of TB treatment prior to starting ART, and some Stage 4/3 conditions may also require a short delay. These subjects will be encouraged to return to initiate ART as soon as they are able, and an appointment will be scheduled if possible. The rapid initiation process will be offered to any subject in the rapid group who returns within four weeks (28 days) of study enrollment. Subjects in the rapid group who request a delay and return more than four weeks later will be initiated under standard procedures, though they will remain in the rapid group for purposes of data analysis.

<u>6d. Standard ART initiation procedures (Track 1 standard initiation group)</u>

Subjects in the standard initiation group of Track 1 will receive standard care as provided by the study site. The right side of Figure 2 illustrates these procedures.

6d i. Determination of treatment eligibility

Following the study site's usual procedures, subjects in the standard group will be asked to give a blood sample for a CD4 count on the day they test positive for HIV. Samples are sent to an off-site laboratory for processing, with results typically available in approximately one week. Patients are then asked to schedule a return visit one week later to receive their CD4 count results and be informed of their eligibility for ART.

For study subjects receiving standard care, with whom the study team will have no direct contact after study enrollment, laboratory results will be monitored to determine those who are treatment-eligible and should be enrolled in the study. No further data will be collected for subjects who are not eligible for treatment. Follow up of those who are eligible for treatment will be by passive record review only, as described below.

6d ii. Remaining ART readiness and initiation steps

Subjects who are eligible for treatment will follow the site's standard procedures for initiating ART, based on South African guidelines. These include 1-3 return visits to the clinic for remaining laboratory tests (creatinine, full blood count, ALT, pregnancy, syphilis), TB diagnosis and treatment if required, adherence counseling, wellness education, and a physical exam. Medications will then be prescribed by the site doctor or primary health care nurse and dispensed by the site's pharmacy.

6e. Follow up

6e i. Direct study interaction

Direct study interaction with Track 1 study subjects will cease as follows:

- *Rapid group (Track 1)*: upon completion of the ART initiation steps described above. For most subjects, this will be at the end of their initial visit, though some may return a second time to complete the initiation procedures (e.g. patients with TB or those requesting a delay).

- *Standard group (Track 1)*: after randomization, at which point subjects will be managed by the study site following standard procedures.

From these points on, all subjects, regardless of which type of initiation they received, will be managed by the site following its routine procedures for pre-ART and ART care. Because this study evaluates routine practice and uses retention in care as an outcome, any further study interaction with subjects would affect the results, in particular loss to follow-up. There will therefore be no post-baseline study visits, only routine care visits not involving study personnel. Standard procedures will also be implemented by the study site (not the study team) for study subjects who miss scheduled visits.

Under current guidelines[41], adult ART patients return for routine clinical consultation visits in months 1, 2, 3, and 6 (and six-monthly thereafter). During these visits, patients have a medical exam and receive a supply of medications. All medications will be provided by the treatment site under usual treatment guidelines. A routine viral load measurement is conducted at the 6-month visit. Patients must typically return to the clinic every 1-3 months for medication pickups, whether or not they are due for a clinical consultation.

6e ii. Medical record follow up

Study subjects will be followed through review of routine medical records kept by the study site for a minimum of 10 months after the date of enrollment in the study for Track 1. This will allow all subjects who initiate ART within 3 months of study enrollment to reach the point at which the routine 6-month viral load is called for, with an additional month allowed for the viral load test to be completed. Subjects will continue to be followed until the final date of study data censoring, up to a maximum of 24 months after study enrollment.

In the study team's experience at other clinics in South Africa, a few patients who remain in care and make a clinic visit at the point at which the routine 6-month viral load test should be done do not have viral load results reported in their medical records. Should this happen, the subject's paper file and laboratory records at the site will be reviewed to confirm that no viral load test was done. If no test was done, a note will be placed in the subject's clinic file to remind clinic staff to ask the subject to provide a sample for viral load testing when she or he next comes to the clinic for routine medication pickup, which typically takes place every 2-3 months.

A blank consent form addendum will be placed in each Track 1 subject's clinic file to be used if the subject's care is being transferred to another clinic. The addendum will request consent to collect medical record data during the follow up period from the new clinic. It will be administered by study staff only if a transfer is being processed.

<u>6f. Retrospective comparison group</u>

As a second comparison group for Track 1, in addition to the standard group, data will be collected retrospectively for a sample of patients who were determined to be eligible for ART at Thuthukani Clinic during the 12 month period preceding the start of Track 1 enrollment. As there were no major changes to South African treatment guidelines during this period, the retrospective comparison group will provide information about uptake of ART before any study-related changes were made at the clinic.

Eligible subjects for the comparison group will be selected from the HIV counseling and testing (HCT) register maintained by the study clinic. This register indicates patients newly found to be ART-eligible from among those receiving an HIV test or a pre-ART CD4 count. Patients who became eligible over the period April 2012-March 2013 will be enrolled consecutively, starting with the later date and working backward in time until the target sample size is reached. Follow up data on pre-ART care, ART initiation, retention on ART, and viral suppression at 6 months will then be collected from the site's ART clinic files and electronic medical record system. Each subject will be followed for a 10-month period from the date at which the clinic determined that the patient was eligible for ART, corresponding to the 10-month primary outcome indicator for Track 1. A temporary identifier linking the HCT register record to the follow-up records will be created and retained until all data pertaining to an the individual study subject have been collected and quality checked. Because all data will already exist at the time the sample is selected, the identifier will serve only to link records at the same site, and all records can be accessed on site, at the same time, the identifier for any individual subject will be kept for a very short period.

Procedures for collecting data for this group will be similar to those used for the Track 2 retrospective group, described below. The study will have no direct interaction with subjects in this group.

7. Procedures for Track 2

Track 2 of the study is a pre/post observational evaluation of immediate treatment initiation for pregnant women. New national guidelines introduced in South Africa in April 2013 call for ART to be initiated immediately, at the first antenatal clinic visit, for all pregnant women regardless of CD4 count. This guideline change provides an opportunity to evaluate the impact of rapid ART initiation for this population using an observational approach, by comparing treatment outcomes before and after the change was implemented. The much simpler study design required for a pre/post observational study is described below.

7a. Procedures—prospective (post) group

7a i. Identification of potential subjects and informed consent

The study will enroll HIV-positive pregnant women not yet on ART. At the study site, the site's staff will inform such women about the study immediately after routine post-test counseling or, for patients already known to be HIV-positive, at their first antenatal visit. The site staff will explain that a study is underway and ask if the patient wishes to learn more about it. Patients who indicate that they may be interested in participating in the study will be referred to a study assistant. Those who do not wish to participate will proceed with a standard clinic visit.

Site counselors who perform the pre-screening will record in a notebook patients whom they do not refer for screening, to allow the study to collect aggregate statistics about the overall patient population and compare it to the screened population. Fields that will be recorded in this notebook are date, age in years, gestational age in weeks, and primary reason for not being screened. (We anticipate three main reasons: patient's emotional condition; patient's physical condition; refusal. An "other" field will allow the counselor to record any other reason.) No identifiers of any kind will be collected in this notebook, which is intended to provide only aggregate information.

Upon referral to the study assistant, patients will receive a more complete description of the study, including why it is being done, the steps involved, and the need for written informed consent. They will be assured that participation is voluntary and that they can withdraw from the study at any time, without affecting the quality of care provided by the site. They will also be offered the opportunity to ask questions. They will then be asked to provide written informed consent to participate.

The study assistant will complete a short screening form to record details about each patient screened. The screening form will not collect any identifiable information pertaining to individual patients prior to receipt of written informed consent.

7a ii. Questionnaire

After a subject consents to be in the study, the study assistant will administer the same questionnaire as used in Track 1, documenting the subject's basic demographic and socioeconomic characteristics, primary reason for seeking an HIV test or HIV care, and costs incurred per clinic visit. Height and weight will be recorded to help assess balance between the two study groups (pre and post). Additional questions will be added to the questionnaire to record basic information about the current pregnancy, including estimated gestational age, previous exposure to antiretroviral drugs for PMTCT, and number of other children.

Following administration of the questionnaire, study subjects will be thanked and escorted back to the clinic's ANC staff to continue a routine antenatal visit.

7b. Procedures—retrospective (pre) group

Subjects for the Track 2 "pre" group (pregnant women offered standard care by the site before the guideline change) will be selected from the site's PMTCT register. Subjects who meet inclusion/exclusion criteria will be enrolled consecutively up to the maximum sample size for this component of the study is reached. Data collection for these subjects will be by record review only.

7c. Follow up

Direct study interaction with Track 2 study subjects will cease as follows:

- *Prospective (post) group*: after administration of the questionnaire, at which point subjects will be managed by the study site following standard procedures.

- *Retrospective (pre) group*: no direct interaction at any time during the study.

7d. Record review

Study subjects will be followed through review of routine medical records kept by the study site for a minimum of the remaining months of pregnancy prior to delivery. Subjects will continue to be followed in the medical records after delivery until the final date of study data censoring, up to a maximum of 24 months after study enrollment.

Delivery information (date and outcome of delivery) that is not found in the study site's own records will be sought at one of the maternity ward facilities to which women are regularly referred for delivery, Tembisa Hospital and Esangweni Clinic. A letter indicating the approval of these facilities for this limited data collection will be submitted to the IRB prior to data collection. At these facilities, the delivery ward register and/or patient files will be used to fill in missing fields in study subject records. No additional data fields beyond what are already included in the consent forms and CRFs will be collected from these facilities. This procedure is necessary to address the problem of information that is supposed to be recorded in the study clinic files but is missing due to incomplete communications between the study clinic and delivery facilities.

8. Data Collection and Management

8a. Sources of data

The study will collect data from four sources, as follows:

1. Screening form. Screening forms will be completed to confirm study eligibility and consent.

2. Case report form. The study nurse will use the CRF to record all steps taken by the patient and provider during the period of study interaction. The CRF will include a short questionnaire to be administered to all subjects upon consent.

3. Medical records. Routinely collected medical record data will be abstracted from medical records maintained by the study site in electronic and paper format.

4. Pre-screening notebook. Aggregate statistics about the number of potentially eligible patients not screened for the study and the reasons for not screening will be calculated from notebooks used by clinic counselors.

<u>8b. Screening form and pre-screening notebook</u>

The study assistant will complete a screening form for each patient referred after pre-screening. The screening form will record whether the patient meets each inclusion and exclusion criterion, provides written informed consent, is eligible for ART, and is eligible for study enrollment. For patients enrolled, the Study ID number and medical record number will be added to the screening form, which will become part of the case report form for study subjects. Track 1 and Track 2 will have different screening forms.

Study counselors will enter a row in a pre-screening notebook for patients not referred for screening. This will allow aggregate information about the overall patient population to be collected and help determine whether those who were not screened differed from those screened. Data will be on sex, age in years, and reasons for not being screened. No identifiers will be recorded or collected.

<u>8c. Case report form</u>

The study nurse will be responsible for completing a case report form for each study subject. The CRF will record consent, meeting of inclusion and exclusion criteria, enrollment, study group, and study identification number for all subjects. For Track 1, it will also record completion and results of lab tests, physical exam, and educational/counseling session; the decision to dispense ARVs; and any other information pertinent to the subject's participation in the study. This form will also record the dates and times (hour: minute format) at which each subject enrolled in the study, completed intermediate steps, and was dispensed ARVs. The CRF is expected to be completed (fully filled out) by the end of the day on which the subject is initiated on ART.

For those offered standard initiation, the CRF will record treatment eligibility when that information becomes available (usually upon receipt of CD4 count results from the off-site laboratory). No further information will be entered into the CRF for these subjects, as follow-up from this point on will be by record review only.

The CRF will include a short, closed-ended questionnaire to be administered in both tracks by the study assistant after consent. The questionnaire will elicit basic information about:

- Demographic characteristics of patient and patient's household (marital status, household composition, duration living in current location)
- Socioeconomic characteristics of patient (employment status, income level)
- Costs to patient of obtaining care at site (transport costs, loss of income, etc.)
- Primary reason for seeking an HIV test (symptoms, pregnant, partner diagnosed, concern about personal risk, etc.), history of previous HIV tests, household members with HIV/on ART, previous use of the study clinic

The last page of the CRF will include identifying information about the subject, including name, date of birth, and national identification number. This information will not be entered into the study database but will be used only for matching CRF and medical record data. This information will be collected for both study tracks (in Track 2, prospective group only), as matching CRF data to medical record data will be necessary for both tracks.

Only some components of the CRF will be used for subjects in Track 2 (questionnaire, receipt form, identification form), not the entire form.

8e. Medical record data

Most data for the study will be collected from the site's routinely maintained electronic medical records, which are maintained in an information system called Therapy Edge-HIV™. Records in this information system are typically only created once a patient starts ART. Paper-based medical records kept by the site will therefore be used to supplement the electronic medical record system as needed. The fields to be collected are listed below.

To be used to link electronic and paper-based records to study identification number only:

- Electronic medical record system number
- Clinic file numbers
- National ID number
- Name
- Date of birth

All subjects at baseline (ART initiation):

- Date of positive HIV test (if available)
- Sex
- Age in years
- Weight and height or BMI
- Date treatment eligibility determined, if reported
- Date and result of eligibility-determining CD4 count
- Date and result of TB symptom screen
- Date, type, and result of TB test(s)
- TB treatment, if relevant
- WHO stage and clinical conditions
- Date ART initiated (first dose of drugs dispensed)
- First-line regimen prescribed

For all clinic visits for duration of study follow up, including initial visit and pre-ART visits (to be used to estimate resource utilization):

- Date of visit
- Primary reason for visit (e.g. scheduled medical visit, medication pickup, unscheduled medical visit)
- Types of professionals seen (e.g. nurse, doctor, pharmacy assistant, counselor)
- Group and individual sessions attended (e.g. wellness course, adherence education)
- Numbers and types of all lab tests conducted (including CD4 counts and viral loads)
- Other procedures (e.g. x-rays) performed

- Medications prescribed and dispensed (ARV and non-ARV)
- Admissions for inpatient care since previous visit, if recorded
- Details of inpatient care received (facility, number of days, medications, lab tests, etc.) if recorded

At interim and final dates of data censoring:

- Date and result of most recent viral load test
- Status at clinic (in care / died / transferred to another site / stopped care / lost to follow up, defined as > 1 month late for last medication pickup)
- New or recurring HIV-related conditions
- Date and cause of death if applicable
- Date and result of most recent CD4 count

Track 2 subjects only (pregnant women):

- Gestational age at diagnosis or first ANC visit for current pregnancy if previously diagnosed
- Prior exposure to PMTCT
- Date of delivery
- Medication pickup dates and quantities of ARVs dispensed
- Number of weeks on ART at delivery

Paper-based medical records maintained by the study site will be accessed when necessary to confirm or supplement information in the electronic medical record system. Paper records and registers will be used for subjects in the retrospective group of Track 2, who will initially be selected from the PMTCT register, and for the retrospective comparison group of Track 1, who will initially be selected from the HCT register. Paper files and clinic registers will also be used if it is found that routine data entry into the electronic medical record system is incomplete or backlogged. Paper medical records or records kept in a different electronic medical record system will also be used for patients who transfer to another clinic during the study follow up period and consent to have their new clinic's records accessed.

8f. Data management

8f i. Study identification numbers and linking of records

Upon consenting to participate in the study, each subject will be assigned a random study identification number. This will be a five-digit number in which the first digit indicates the study track (1 or 2). The remaining four digits will be a randomly generated number that does not provide any identifying information.

A password-protected, encrypted linking file will be created in Excel to link study identification numbers to subjects' names, dates of birth, national identity numbers, and clinic file numbers. These fields are needed to ensure that study ID numbers can be correctly matched to the identification numbers used in the site's electronic medical record system (MRS) where patient records are kept after ART initiation. When a subject has been matched to a record in the MRS, the MRS record number will also be added to the linking file. The linking file will be a separate document from any other study files. Access to the linking file will be limited to the study team, and the file will be stored in a secure location separate from the study database.

8f ii. Data management and storage

Study specific data will be captured on site on the paper case report form described above. These forms will be scanned on site by study staff. Scanned files will immediately be password protected and encrypted. Protected and encrypted files will then be transferred at the end of each day or at weekly intervals to an access-limited Dropbox folder and then immediately downloaded to the study team's secure office network. Upon confirmation of receipt of the scanned files, the original pdfs will be deleted from the study computer on site. Original paper case report forms will be physically transferred to the study office once a week and stored in a secure cabinet. (Source documentation such as printouts of results of laboratory tests will remain at the study site and filed following the site's usual record-keeping procedures. Source documents will never be removed from the study site.)

Study-specific data from case report forms will be entered and managed using Research Electronic Data Capture (REDCap, http://project-redcap.org), a web-based tool for electronic collection and management of research data. The REDCap system provides a secure, web-based platform for data entry that allows real time monitoring, querying, and quality control of data by an off-site data manager. It allows access to data to be assigned and restricted to individual study staff as needed and creates audit trails to monitor appropriate access. It is supported by the Boston University IT office (https://redcap.bumc.bu.edu/). It has previously been used successfully in South Africa, where the University of the Witwatersrand is a REDCap Consortium partner.

The study's lead data analyst will review the study database in REDCap on a daily basis during the pilot phase and on a weekly basis (or more frequently) thereafter. The data analyst will run data cleaning routines regularly and generate queries to return to the site-based staff for resolution as needed.

Medical record data for study subjects will be downloaded from the site's existing electronic medical record system, as described above. Records in this system are created only when patients initiate ART, and thus do not capture pre-ART care (HIV tests, monitoring CD4 counts, etc.). The lead data analyst will be responsible for ensuring that study identification numbers are correctly matched to the record numbers in the MRS. In instances where the data analyst cannot match a study ID number to an electronic medical record number using only the fields in the study linking file, a query will be sent to the on-site study staff, who will access the clinic's paper-based patient files to determine whether the subject ever initiated ART and, if so, locate the correct MRS number.

For subjects in the standard group, eligibility for study enrollment can only be determined once treatment eligibility is established by CD4 count or other criterion. For these subjects, the study team will review the site's paper-based patient files on a monthly basis to determine whether and when the subject has been found eligible for treatment and if treatment has been initiated. This information will be entered onto case report forms and captured in the REDCap database as described above.

A note or tab indicating that a patient is a participant in the study will be attached to the cover of each subject's paper medical record at the study site, to assist site and study staff in locating study subjects' files should this be necessary and to ensure that site staff are aware of which patients are study participants.

8f iii. Data quality assurance

As case report forms are completed, they will be quality-checked by a member of the study team to ensure missing or illogical data are corrected. If any changes to paper forms are made, the original data will be left and crossed out, and the corrected data will be written next to it with a signature and date of the person who made the change. As described above, the lead data analyst will review subject records in REDCap on a weekly basis or more frequently if needed so that queries can be sent to the on-site staff promptly. The lead data analyst will also be responsible for data cleaning and management. Data completeness and quality will also be reviewed on a monthly basis by the investigators. Monthly updates on study enrollment numbers and randomization allocation will be sent by the analyst to the PI and co-investigators for monitoring progress.

8f iv. Aggregate statistics

Subjects who consent to study participation but are not eligible for ART and those who are found to be study ineligible for any other reason will not be enrolled in the study, as explained above. These subjects' ineligibility for the study will confirmed by the lead data analyst. Data already collected for these subjects will be used to compile aggregate information on a monthly basis regarding patients at this site who are not eligible for treatment (e.g. average age and average CD4 count of patients who test HIV-positive). Individual data will be destroyed at the end of each month following compilation of the monthly aggregate statistics. Source documents for any test results (CD4 count, TB test result) generated during screening procedures will be retained in the study clinic's file for the patient.

8f v. Destruction of data

Data will be retained in secure study databases for 7 years following the completion of the study, including completion of all data analysis and report writing associated with the study and closing of the IRB-approved protocol. Study data sets and other electronic files containing study data will then be deleted from electronic storage locations and hard copies of study documents will be shredded.

9. Staff Supervision and Training

The study will be implemented by a Johannesburg-based team employed by the Health Economics and Epidemiology Research Office (HE^2RO), a division of the School of Clinical Medicine at the University of the Witwatersrand. HE^2RO has collaborated closely with the Principal Investigator and other Boston University co-investigators for nearly ten years and completed numerous studies of relevance to the RapIT study.

The local study team will be trained and supervised by the Principal Investigator for HE^2RO. The local team will include a study nurse with responsibility for implementation of all study procedures at the site, one or two study assistants, a data capturer, and a data manager. The study nurse will have the training and qualifications required to initiate ART and be authorized to do so under South African guidelines. Study assistants, who will administer the questionnaire and deliver educational sessions on treatment and wellness, will also be trained as HIV pre- and post-test and ART adherence counselors.

All members of the study team will be trained in and adhere to the standards laid out in the Belmont Report and NIH guidelines for research and in research ethics applicable to South Africa. Human subjects ethics training will be repeated on an annual basis to ensure that the study team's understanding of research ethics is current.

10. Sample Size

Track 1: In a recent study we conducted in Johannesburg, we found that among patients eligible for ART, 46% initiated ART within 16 weeks of eligibility[14]. In an analysis of data from the same Johannesburg site, we estimated that among more than 3000 patients initiated on ART in 2010 and 2011 who remained in care, 77% were virally suppressed at the routine 6-month viral load. We would thus expect approximately 0.46 x 0.77 = 35% of those eligible to have both started treatment and be virally suppressed by the 6-month viral load. For our study site we conservatively estimate that 50% of those eligible for treatment in the standard group will be suppressed by 10 months after their HIV test. We anticipate that the rapid initiation strategy will increase the proportion suppressed to 70%.

For our primary analysis, using an α of 0.05, power of 90%, 1:1 randomization, and an uncorrected Fisher's exact test, we would need to enroll 124 HIV positive ART-eligible subjects per group (248 total). We expect 70% of all HIV-positive patients to be eligible for ART under current South African guidelines on the day they test positive for HIV or make their first HIV care visit, based on results from published studies in South Africa[13,19]. Therefore to reach 124 subjects per arm who are treatment eligible on the day of testing we will need to consent 177 subjects per group (354 total). To allow stratification in the analysis by sex and by site, we will consent up to 300 per group (600 total) to have adequate power. We will enroll in the study up to 67% of this total (enrollment=400).

The sample size for the Track 1 retrospective comparison group will be 120. This group will be limited to patients already found to be eligible for ART at one of the two study sites (Thuthukani). They will be compared with each of the two randomized prospective groups (rapid and standard). We expect that Thuthukani will account for approximately 60% of total prospective enrollment of 400, or 120 in each of the randomized prospective groups. We will thus aim to enroll the same the number of subjects into the retrospective comparison group as in each prospective group.

Track 2: In a study we recently conducted among pregnant women eligible for ART on the day of testing positive for HIV at a different site in Johannesburg, South Africa, we found that 77% of those who initiated ART prior to delivery returned for at least one clinic visit post delivery, which that study used as a proxy to indicate retention on ART through delivery. We believe that an improvement of 25%--i.e., an increase in retention through delivery from 77% to 96%--would be operationally meaningful.

For our primary analysis, using an α of 0.05, power of 90%, 1:1 allocation of prospective and retrospective subjects, and an uncorrected Fisher's exact test, we would need to enroll 76 HIV positive ART-eligible subjects per group (152 total). If the rate of enrollment allows, we will enroll up to twice this many subjects (305 total) to allow the analysis to be stratified by starting CD4 count, which was previously a criterion for treatment initiation in pregnant women.

Pilot: The first 20 subjects consented for the study in Track 1 and the first 5 subjects consented in the prospective group of Track 2 will serve as a pilot phase to ensure that study procedures run smoothly and confirm that study staff are fully trained. Subjects in the pilot phase will receive the same study procedures as described above for their track and group, and their

data will be included in the analysis. Pilot subjects will be included in the enrollment totals for each track.

The total sample size for the study will therefore be 905 screened and 705 enrolled, as shown below:

**Track**	**Total screened**	**Consented**	**Record review only**	**Screened and not enrolled (not eligible for ART)**	**Enrolled**
Track 1	720	600	120	200	520
Track 2	305	153	152	0	305
Pilot phase	n.a.	n.a.	n.a.	n.a.	n.a.
**Total**	905	753	152	200	825

11. Outcomes and Data Analysis

11a. Outcomes

11a i. Primary outcome

Track 1: The primary outcome for Track 1 is the proportion of subjects in each group alive, in care and virally suppressed at the routine six-month on ART monitoring visit within 10 months of a positive HIV test or initial HIV care visit if previously diagnosed. The analysis period will start at study enrollment and continue through the earlier of the patient's six-month viral load or 10 months after the patient's HIV test.

Under South African treatment guidelines, all ART patients have a monitoring viral load done at six months after ART initiation. We will use this measure to assess our primary outcome. In order to create comparable follow-up time in both groups and to allow all subjects enough follow up time to have a six-month viral load, for the primary outcome analysis we will consider all ART-eligible patients who do not have a six-month viral load for any reason within 10 months of study enrollment to be failures. Viral suppression will be defined as <50 copies/ml. Patients in the rapid group who do not initiate treatment on the day of their HIV test will remain in the intervention arm for analysis (i.e. intention to treat).

Our primary outcome will be measured 10 months after study enrollment because the first 6 months on ART is the time when patients are most likely to be lost or die[42,43]. At the 10-month point after study enrolment, all subjects who started ART within 3 months of enrolment will have had the opportunity for at least 6 months of exposure to treatment and allow an additional month for subjects to return to the clinic for their routine 6-month viral load test. If rapid initiation triggers a higher rate of loss to follow up after starting treatment, the impact is likely to be evident within six months of initiation. Six months of ART is also sufficient to achieve viral suppression in the vast majority of patients. In an analysis of >13,000 patients who initiated ART at a large clinic in Johannesburg, 75-80% of all patients were virally suppressed after a median (IQR) of 3.9 (3.7-5.0) months[44]. We would thus expect at least 80% of patients to reach viral suppression by 6 months on ART.

Track 2: The primary outcome for Track 2 is the proportion of subjects in each group who adhere to ART or the prior PMTCT regimen until delivery. The number of weeks a pregnant woman is on ART before delivery is the most important predictor of perinatal HIV transmission[7]. The guideline change to immediate ART initiation for pregnant women is likely to lead to earlier initiation for most pregnant women, but the intervention will be effective only if patients adhere to ART through the duration of pregnancy. Adherence will be measured as making monthly medication pickups to allow a continuous supply of ARVs through delivery. The analysis period for the primary outcome will start on the day of study enrollment, which is the date of a positive HIV test or the first antenatal visit of the current pregnancy, for women previously diagnosed, and end at the actual or estimated delivery date.

11a ii. Secondary outcomes

Secondary outcomes will include:

1. Average cost per patient who is alive, in care, and virally suppressed within 10 months of study enrollment (Track 1).
2. Cost per patient adhering to ART through delivery and the cost per infant infection avoided, using estimated vertical transmission rates (Track 2).
3. Proportion of subjects in each track and group alive, on ART, and virally suppressed at six-month intervals and the final date of data censoring.
4. Average time to ART initiation (days) for each track and group.
5. For Track 2, average gestational age at ART initiation (weeks) and average duration (weeks) on ART prior to delivery.
6. For Track 2, if enrollment numbers allow, proportion of subjects with CD4 count > 350 and ≤ 350 achieving the primary outcome of retention on ART (or PMTCT regimen) until delivery.
7. Patient-level predictors of treatment uptake, retention in care, and viral suppression.
8. Prevalence of TB symptoms and confirmed TB, time to initiation of TB treatment, and time to initiation of ART among patients with TB.
9. Process indicators including:
   - Acceptance of rapid initiation strategy (% of patients offered rapid initiation who accept)
   - Average time elapsed (days) between HIV test and dispensing of first dose of ARVs
   - Average cost to patients of initiating treatment, including travel and other out-of-pocket costs and time spent in clinic

All outcomes will be estimated for each study population and compared between the two study populations where relevant.

11b. Clinical outcomes analysis

For both tracks and for primary and secondary clinical outcomes, the analytic approach for clinical outcomes will be the same. All analyses will be by intention-to-treat: subjects will be analyzed according to the intervention they were supposed to receive, whether or not they adhere to the defined intervention.

The analysis will begin with a simple comparison of the two treatment groups with respect to baseline predictors of outcomes. We will then conduct a crude analysis comparing the proportion of patients achieving each dichotomous outcome by group and crude risk ratios and 95% confidence intervals

stratified by group. For average gestational age at ART initiation and average time on ART before delivery for Track 2, we will calculate the mean difference between groups and corresponding 95% confidence interval.

Since adherence has been a particular concern for pregnant women who were not eligible for ART under the previous guidelines, the primary outcome for Track 2 will be stratified in secondary analysis by CD4 count at the time of initial ANC visit if enrollment numbers by the end of the study period are large enough to allow a stratified analysis. Subjects in the prospective group found after ART initiation to have CD4 counts above 350 will be compared to those in the retrospective group who were offered AZT under the previous guidelines, while subjects in the prospective group found after ART initiation to have CD4 counts below 350 will be compared to those in the retrospective group who were offered full ART under the previous guidelines.

Because we will not be randomizing large numbers of patients in Track 1 and because no randomization will occur in Track 2, it is possible that some baseline covariates may be imbalanced by treatment arm. To adjust for potential differences by baseline covariates, a log-linear regression model will be used to estimate adjusted risk ratios and a linear regression will be used to adjust mean differences.

Variables considered to be important in the univariate stage will be included in multivariate models. Adjusted analyses will include covariates which are unevenly distributed across groups and which could plausibly affect viral suppression or time to ART initiation. These potential confounders include demographic (e.g. age, sex) and socioeconomic (education, distance from clinic, employment, etc.) variables, medications, baseline CD4 counts, prior receipt of PMTCT, and low BMI, among others. Each of these models can then be used to look for patient-level predictors of treatment uptake, viral suppression, and retention in care.

<u>11c. Cost outcomes analysis</u>

At the relevant endpoints (10 months after study enrollment for Track 1, delivery for Track 2, and other dates of data censoring), all resource usage and outcome indicators will be extracted from subjects' medical records and case report forms. Unit costs will be obtained from external suppliers and the site's finance and procurement records and applied to the resource usage data to provide a cost per study patient. Costs will be measured from the provider perspective and will include the cost of all resources utilized for each study subject, including drugs, laboratory tests, outpatient visits, and fixed costs such as building space, equipment, and management staff. We will estimate the average cost to the provider per patient achieving viral suppression by 10 months (Track 1) or adhering to ART until delivery as indicated by monthly medication pickup (Track 2). We will also estimate the average cost per other outcomes achieved, such as per patient remaining in care by specified endpoints. The average cost per outcome will then be compared between rapid and standard initiation groups to provide an estimate of the cost-effectiveness of the two strategies. Costs will be reported as means (standard deviations) and medians (IQRs) in rand and dollars.

<u>11d. Analysis of process indicators</u>

In order to help inform treatment providers and policy makers of the implications of rapid ART initiation in terms of patient acceptance, impact on staff, laboratory work flow, and other patient-level and health systems factors, we will generate descriptive statistics for process indicators for both initiation strategies, including:

- Acceptance of rapid initiation strategy (% of patients offered rapid initiation who accept and frequency of reasons for refusals where provided)
- Mean time elapsed (days) between HIV test and dispensing of first dose of ARVs
- Mean time (hours: minutes) spent in clinic by patients receiving rapid and standard initiation, based on recorded start and end time for each clinic visit.
- Mean cost (rand) to patients of making the clinic visits required for treatment initiation, including transport fares, lost wages, and other costs reported in the baseline questionnaires.

11e. Dissemination of findings

The results of this study will be disseminated as widely as possible in South Africa, where new strategies for reducing pre-ART loss to follow up and promoting earlier treatment initiation are eagerly sought, and in other countries facing similar challenges. A full report will be made to the study site and other local stakeholders and will be circulated widely and posted on our website. We will also develop a short briefing document to send to the national and provincial departments of health, donor agencies, and other interested organizations, and we will present the results at relevant conferences in South Africa and internationally. One or more journal manuscripts will be submitted to an appropriate peer-reviewed international journal. Finally, a flier summarizing the results in lay language will be distributed to patients at the study site and posted in patient waiting areas, to ensure that study subjects and others gain access to the findings. (The flier will be submitted for IRB approval prior to distribution.)

12. Human Subjects Considerations

12a. Risks and protection against risks

For this study, we will not collect any biomedical samples that would not be collected as part of routine care, nor will we implement any clinical procedures that are not routinely carried out under standard of care. The rapid test technologies to be used in this study require exactly the same biological samples (blood, sputum) that would be required for routine care, and the technologies have all been validated and approved for use in South Africa. The strategy being evaluated merely accelerates the timing of events. All biomedical data for the study will be drawn from routinely collected medical records, including patient charts and laboratory reports. The only other new data generated for the study will be responses to questionnaires. We therefore believe that our study poses no physical risks to subjects beyond those routinely encountered. It may, however, pose risks associated with loss to HIV care, emotional distress, breach of confidentiality, and time loss.

12a i. Loss to HIV care

Risk: The study aims to initiate on ART patients who might otherwise be lost to HIV care. Some patients lost before or after ART initiation will seek care at other sites, but many will not and will postpone or end all HIV care and treatment, some until they become more seriously ill, others forever. Although the rapid initiation strategy is expected to increase the proportion of patients who do initiate ART, there is a possibility that patients who accept rapid ART initiation will subsequently discontinue treatment at a greater rate than do those who receive standard ART initiation. Preliminary studies in South Africa suggest that the loss of treatment-eligible patients before starting ART, which is expected to decrease as a result of the intervention, exceeds loss after initiation. It is also likely that patients who discontinue

treatment soon after rapid initiation may never have started at all, had only standard initiation been offered. We therefore expect the intervention to have a net positive impact on our primary outcome, the proportion of patients who are alive, in care, and virally suppressed 10 months after testing HIV-positive or making their first HIV or ANC care visit. We cannot be certain of this result in advance, however. Because this is an evaluation of routine practice and retention in care is a study outcome, no efforts will be made to retain patients in the study, beyond those already routinely undertaken by the study site clinic as part of standard of care.

Protection: As patients who are subjects of this research will be both those who remain in HIV care and those who drop out, and as the research team will have access to information about which subjects are lost, efforts must be made to return these patients to HIV care. However, since losses from HIV care are a primary outcome of this study, study staff cannot interfere with usual patient care. Upon enrollment in care at the site, patients are routinely asked to give a cell phone number and address where they can be contacted at and identify an acquaintance who can be contacted should they not be able to be reached through the previously listed means. The study site maintains a small team of loss-to-follow-up counselors trained in confidentiality and ethical issues around contacting patients. These counselors do not always have access to timely information about which patients have been lost, however. As we will be generating this data, we will work alongside to the counselors to make sure they have adequate, up-to-date information on study subjects' status and can attempt to contact the patients using standard clinic procedures if necessary. In particular, we will ensure that all patients defined as lost to follow up at their 6-month viral load monitoring visit (or by 10 months after study enrollment) are referred to the counselors to be contacted.

12a ii. Emotional distress

Risk: By necessity, the study population for this study will include patients who have been newly diagnosed with HIV. Interacting with them in order to explain the study and confirm eligibility before requesting written informed consent may cause some emotional distress for some potential subjects.

Protection: To minimize any emotional distress caused by interacting with study staff or enrolling in the study, we will train the site's staff to emphasize when they introduce the study to potential subjects that referral to study staff and enrolling in the study are completely voluntary and that those who do not wish to enroll will receive exactly the same care as the study site would otherwise have provided. Potential subjects will also be told that they can discontinue participation even after consenting and can revert to standard, non-study care at the study site at any time. Study staff will be trained to look for signs of emotional distress and instructed to terminate or postpone the enrollment process if subjects appear distraught.

12a iii. Accidental disclosure of HIV status or other loss of confidentiality

Risk: A potential risk of the study concerns the confidentiality of data about HIV status and treatment program enrollment. A high level of stigmatization continues to inhibit the disclosure of HIV status in the study population. Data collected in interviews or from clinic

records or the site's electronic database could be disclosed and reveal a person's HIV status. Additionally, the shopping vouchers received for study participation may cause others to assume participants are part of an HIV-related study if they are used at the store nearest to the clinic.

Protection: Several steps will be taken to protect subjects against the risk of accidental disclosure of HIV status. Individual documents (including signed consent forms, case report forms, and the linking file) which could associate patients with an HIV study will be kept strictly confidential. Signed consent forms will be stored in locked cabinets away from the study site, with access limited to the senior investigators. Paper case report forms will be stored separately from consent forms in a locked cabinet at the study team's office, which is itself located in a secure facility, or in a secure archiving facility. Scanned copies of the CRFs will not include identifiers and will be password-protected. The linking file will also be password-protected, with access limited to study staff. It will be used only for the purpose of linking study identification numbers with electronic medical records and, when necessary for data verification and completion, with paper-based patient files and source documents.

To protect study subjects from being assumed to be participating in an HIV-related study if they use the shopping voucher at the store nearest to the clinic, participants will be informed of this risk and given a list of other stores at which the voucher is redeemable. Store staff are not informed of the nature of this study and vouchers that participants receive do not differ in any way from shopping vouchers purchased for other reasons.

To protect against other violations of confidentiality, study staff will be trained in expectations that they are not to disclose any information collected in the study to anyone outside the study team. All study staff will be required to pass an ethics certification course, such as the on-line certification offered by the NIH. All participants will be encouraged to contact the HIV testing coordinator, principal investigator, or other staff to report any undesirable conduct associated with the study. These reports will be brought to the attention of the study site and the PI, and appropriate steps will be taken to solve the problems, including reporting to relevant ethical review boards.

12a iv. Time loss

Risk: Subjects participating in the study and assigned to Track 1 rapid initiation will be asked to remain in the study clinic for approximately 4-5 hours to allow all procedures to be completed. This is longer than they may otherwise be required to spend at the clinic and may be inconvenient for some subjects.

Protection: Potential subjects will be informed about the time requirements during the informed consent process. Those who wish to participate in the study but cannot remain in the clinic on the same day for the amount of time required will be able to delay treatment initiation until they find a time that is convenient for them, thus mitigating the inconvenience.

<u>12b. Benefits</u>

12b i. Direct benefits

Study subjects offered rapid treatment initiation will benefit from the opportunity to start ART quickly and with fewer clinic visits and time delays than they would otherwise have incurred. Study subjects offered standard initiation will not receive any direct benefits from the study but will receive the same care that would have been provided in the absence of the study.

12b ii. Indirect (societal) benefits

The study is expected to generate substantial indirect benefits to the subjects. The research we will undertake is expected to reveal whether rapid initiation of ART can improve treatment outcomes at a cost that is affordable for treatment service providers and funding agencies. The results of the study may thus lead to improvements in HIV care and keep patients alive and in care longer. The results will also provide evidence about the feasibility of test-and-treat strategies and thus contribute to efforts to improve HIV prevention.

12b iii. Ratio of benefits to risks

The knowledge to be generated by this study will allow HIV treatment programs to identify whether rapid ART initiation is both effective and cost-effective. This study will address an important gap in the current knowledge around ART initiation and what can be done to reduce the already high proportion of patients who are being lost from care after testing positive. There is currently almost no information available on how to reduce these pre-ART losses. As explained in the previous section, we believe that the risk to subjects in our study is minimal and is outweighed by the indirect benefits and the potential importance of the findings.

<u>12c. Costs and payments to subjects</u>

There will be no costs to subjects for participating in this study.

Subjects in Track 1 who consent to participate and complete the study questionnaire will receive a payment of R100 (approximately $12.50) to reimburse for their time and inconvenience. This payment will be made in the form of a voucher for a nearby supermarket or cell phone air time.

Subjects in the prospective group of Track 2 who consent to participate and complete the study questionnaire will receive a payment of R50 (approximately $6.25) to reimburse for their time and inconvenience. This payment will be made in the form of a voucher for a nearby supermarket or cell phone air time.

Subjects in the retrospective group of Track 2 and the retrospective comparison group of Track 1 (retrospective record review only) will not receive any payment.

<u>12d. Recruitment</u>

Identification of potential subjects will occur during HIV post-test counseling, blood draw for CD4 count, return of CD4 count results, or first HIV-positive antenatal visit. All of these activities are conducted within the study site's VCT area. In the VCT waiting area and the clinic's main reception area, a flier will be posted that informs patients that a study is underway and indicates where to get further information. A short pamphlet will be distributed to patients requesting further information. The flier and pamphlet will be translated into the commonly used languages at the study site.

The site's post-test counselors, nurses, and registration staff and the study assistant will inform potentially eligible patients about the study while they are awaiting and/or receiving services. They will explain that a study is underway and that patients who voluntarily consent to participate in the study and are found to be eligible for ART may have the opportunity to initiate treatment immediately, rather than following the standard schedule of visits. Patients who indicate that they may be interested in participating in the study will be referred to the study assistant immediately upon completion of the regular post-test counseling, CD4 blood draw, or return of CD4 count results. Those who do not wish to be referred to the study assistant will receive the site's standard pre-ART care.

12e. Informed consent

Written, informed consent will be sought from all study subjects in Track 1 and the prospective group in Track 2. The informed consent information sheet will describe the nature and goals of the study and assure subjects their information will be kept confidential. For Track 1, it will explain to subjects the possible treatment options in the study:

- The consent form will explain that patients who consent to participate in the study but are found to be not yet eligible for ART will not be enrolled in the study. They will be provided with standard pre-ART care by the study site instead.

- For Track 1, the consent form will explain that patients will not get to decide which group they will be in, but rather that we will decide by chance and they will have a 50-50 chance of being in either group. The form will explain that if they are randomized to the group how their care will be different from standard care. It will also explain that any individual patient can choose not to participate in the study and be offered standard initiation.

- For Track 2, the consent form will explain that the study is limited to a questionnaire and medical record review.

- Consent forms will explain about follow-up through medical record data collection.

The consent form will be administered by a trained study assistant and will indicate that the subject gives us permission to access their clinic records. The consent form will also state that the subject agrees to be interviewed using a baseline questionnaire. Participants will be assured that data collected for our study will be kept strictly confidential and will never be reported to clinic staff or anyone outside the study team.

The full informed consent information sheet and form will be translated into the most commonly used languages at the study site. The languages for which translation is expected to be required are Isizulu, Sesotho, and Xitsonga. If other or additional common languages are needed, the form will be translated accordingly. Translated forms and attestations of translation accuracy will be submitted to the IRB prior to any use of the forms. Subjects who unable to read and/or sign the consent form due to illiteracy will be asked to provide a thumbprint mark, in the presence of a witness who will also sign the form. Patients who do not speak English or any of the languages listed above will be regarded as unable to provide written informed consent, which is an exclusion criterion for the study, and will not be eligible for study enrollment.

Consent will be sought after study inclusion/exclusion criteria have been met, with the exception of eligibility for ART, which will only be determined after consent.

A blank consent form addendum will be placed in each Track 1 subject's clinic file to be used if the subject's care is being transferred to another clinic. The addendum will request consent to collect medical record data during the follow up period from the new clinic. It will be administered by study staff only if a transfer is being processed.

Informed consent will not be sought for the retrospective group in Track 2 or the retrospective comparison group in Track 1, for whom the study will be a record review only. Data for these groups will be collected retrospectively, with identifiers retained in a linking file only long enough to allow separate source documents (e.g. PMTCT register, HCT register, pre-ART register, and electronic medical record) to be matched for individuals. The study team will have no interaction with these groups.

<u>12f. Protection of confidentiality</u>

Subject data will be captured on paper case report forms and scanned on site. The CRFs will be stored in a locked cabinet in a restricted-access room. Identifiers will be removed from scanned pdf copies of the CRFs prior to transferring the files to the study team's offices. All electronic data files will be password-protected and encrypted, with access limited to authorized study staff. Study subjects will be assigned random study ID numbers upon consent, and data files will contain study ID numbers only, without any other individual identifiers.

A password-protected linking file allowing medical record data to be matched to study-generated data will be maintained in a secure location, with access limited to study staff. Medical record data will be downloaded on a monthly basis. Monthly medical record datasets will include data on all patients at the study clinic, not just those included in the study, as it is not possible to select only study participants prior to downloading. As soon as datasets are downloaded, the linking file will be used to select study participants and link their records with their study identification numbers. All identifiers will then be stripped from the clinical data and records for all patients not enrolled in the study will be deleted.

<u>12g. Data safety monitoring</u>

The proposed study will evaluate the effect on patient outcomes and treatment costs of a one-time change in service delivery strategies. The rapid initiation strategy will abide by South African national treatment guidelines, make few changes to existing procedures beyond accelerating the timing, and make no changes to treatment protocols after ART is initiated.

Data collected during the period of study team interaction with study subjects (while subjects are on site and in the care of the study staff) will be monitored weekly, except during the pilot phase when daily monitoring will be performed. Adverse events that occur during the period of interaction will be reported according to and within the time periods required by IRB policies.

After study interaction with subjects ends (typically upon dispensing of the first month's supply of ARVs), any attempt to follow patients in real time could potentially bias the study results. After study procedures associated with enrollment and ART initiation have been completed, data will only be collected passively, from review of routine medical records. All of the data used by the study that could be used to monitor patient safety after treatment initiation will be routinely collected medical record

data generated by the site itself, not by the study. The study team will have access to these data only after they have been entered into the site's electronic medical record system and then downloaded by the study's lead data analyst, which will happen on a monthly basis. UPs, AEs, and SAEs that occur during this period will therefore only come to the study team's attention at least several weeks after they occur. These will be reported according to and within the time periods required by IRB policies, but can only be reported after the study team becomes aware of the events through review of the downloaded electronic medical record data.

Because there will be no active tracing of subjects who are lost to follow up after study team interaction ends, beyond any routine tracing conducted by the study clinic, the true status of subjects who are lost to follow up will not be known. Loss to follow up will be reported in study monitoring reports but the study team will not be aware of deaths among subjects lost to follow up unless these have been reported to the study clinic by household members.

Since the sample size for the study is not large and the relatively brief duration of recruitment would minimize its impact, a formal data safety and monitoring board will not be employed. Instead, the PI will receive reports from the study site on a monthly basis describing recruitment and the primary outcome by study population and reporting on any unexpected occurrences. An interim analysis will be conducted once, when one half of the study participants in Track 1 complete follow-up (reach the primary endpoint for Track 1). Only if the p-value from unadjusted analyses of the association between randomization group and the primary outcome falls below an alpha of 0.01 in the interim analysis will the study be stopped early.

Further details on data safety monitoring are included in the data safety monitoring plan in the appendix to this protocol.

13. References

1. Fox MP, Sanne IM, Conradie F, et al. Initiating patients on antiretroviral therapy at CD4 cell counts above 200 cells/microl is associated with improved treatment outcomes in South Africa. *AIDS* 2010; 24: 2041–50.
2. Lawn SD, Myer L, Harling G, Orrell C, Bekker L-G, Wood R. Determinants of mortality and nondeath losses from an antiretroviral treatment service in South Africa: implications for program evaluation. *Clinical Infectious Diseases* 2006; 43: 770–6.
3. Lawn SD, Myer L, Orrell C, Bekker L-G, Wood R. Early mortality among adults accessing a community-based antiretroviral service in South Africa: implications for programme design. *AIDS* 2005; 19: 2141–8.
4. Severe P, Juste MAJ, Ambroise A, et al. Early versus standard antiretroviral therapy for HIV-infected adults in Haiti. *NEJM* 2010; 363: 257–65.
5. Cohen M, Chen Y, McCauley M, et al. Prevention of HIV-1 infection with early antiretroviral therapy. New England Journal of Medicine 2011; 365: 493–505.
6. Leisegang R, Cleary S, Hislop M, et al. Early and late direct costs in a Southern African antiretroviral treatment programme: a retrospective cohort analysis. *PLoS Med* 2009; 6: e1000189.
7. Chibwesha CJ, Giganti MJ, Putta N, et al. Optimal time on HAART for prevention of mother-to-child transmission of HIV. *J Acquir Immune Defic Syndr* 2011; 58: 224–8.
8. Rosen S, Fox MP. Retention in HIV care between testing and treatment in sub-Saharan Africa: a systematic review. *PLoS Med* 2011; 8: e1001056.
9. World Health Organization. Global HIV/AIDS response: epidemic update and health sector progress towards universal access. Progress report 2011. Geneva: WHO. Available from: http://whqlibdoc.who.int/publications/2011/9789241502986_eng.pdf.
10. Sanne I, Westreich D, Macphail AP, Rubel D, Majuba P, Van Rie A. Long term outcomes of antiretroviral therapy in a large HIV/AIDS care clinic in urban South Africa: a prospective cohort study. *J Int AIDS Soc* 2009; 12: 38.
11. Boulle A, Van Cutsem G, Hilderbrand K, et al. Seven-year experience of a primary care antiretroviral treatment programme in Khayelitsha, South Africa. *AIDS* 2010; 24: 563–72.
12. Fox MP, Rosen S. Patient retention in antiretroviral therapy programs up to three years on treatment in sub-Saharan Africa, 2007-2009: systematic review. *Trop Med Int Health* 2010; 15 Suppl 1: 1–15.
13. Larson BA, Brennan A, McNamara L, et al. Lost opportunities to complete CD4+ lymphocyte testing among patients who tested positive for HIV in South Africa. *Bull World Health Org* 2010; 88: 675–80.
14. Larson BA, Ndibongo B, Brennan A, et al. Point-of-care CD4 testing after HIV diagnosis to reduce losses to initiation of antiretroviral therapy: an evaluation of a pilot program of Themba Lethu Clinic, Johannesburg, South Africa. Sixth International AIDS Society Conference on HIV Pathogenesis, Treatment and Prevention. Rome, July 17-20, 2011.
15. Stinson K, Boulle A, Coetzee D, Abrams EJ, Myer L. Initiation of highly active antiretroviral therapy among pregnant women in Cape Town, South Africa. *Trop Med Int Health* 2010; 15: 825–32.
16. Katz I, Essien T, Marinda E, et al. Antiretroviral refusal among newly diagnosed HIV-infected adults in Soweto, South Africa. *AIDS* 2011; 25: 2177–81.
17. Myer L, Zulliger R, Pienaar D. Diversity of patient preparation activities before initiation of antiretroviral therapy in Cape Town, South Africa. *Trop Med Int Health* 2012; 17: 972–7.
18. Lawn SD, Brooks SV, Kranzer K, et al. Screening for HIV-associated tuberculosis and rifampicin resistance before antiretroviral therapy using the Xpert MTB/RIF assay: a prospective study. *PLoS Med* 2011; 8: e1001067.

19. Lessells RJ, Mutevedzi PC, Cooke GS. Retention in HIV care for individuals not yet eligible for antiretroviral therapy: rural KwaZulu-Natal, South Africa. *J Acquir Immune Defic Syndr* 2011; 56: 79–86.
20. Rosen S, Ketlhapile M, Sanne I, Desilva MB. Cost to patients of obtaining treatment for HIV/AIDS in South Africa. *S Afr Med J* 2007; 97.
21. Magagula TK, Brennan A, Ive P, Imogen J, Matoe T, Maskew M. A prospective study assessing patient flow at a large public sector HIV/AIDS clinic, Johannesburg, South Africa. SAHARA Conference, Port Elizabeth, South Africa, Nov 28-Dec 2, 2011.
22. Glencross DK, Coetzee LM, Faal M, et al. Performance evaluation of the point-of-care CD4 analyser Pima using capillary blood sampling in field tests in South Africa. *J Int AIDS Soc* 2012; 15: 3.
23. Mtapuri-Zinyowera S, Chideme M, Mangwanya D, et al. Evaluation of the PIMA point-of-care CD4 analyzer in VCT clinics in Zimbabwe. *J Acquir Immune Defic Syndr* 2010; 55: 1–7.
24. Jani IV, Sitoe NE, Alfai ER, et al. Effect of point-of-care CD4 cell count tests on retention of patients and rates of antiretroviral therapy initiation in primary health clinics: an observational cohort study. *Lancet* 2011; 6736: 1–8.
25. Sukapirom K, Onlamoon N, Thepthai C, Polsrila K, Tassaneetrithep B, Pattanapanyasat K. Performance evaluation of the Alere PIMA CD4 test for monitoring HIV-infected individuals in resource-constrained settings. *J Acquir Immune Defic Syndr* 2011; 58: 141–7.
26. Jani IV, Sitoe NE, Chongo PL, et al. Accurate CD4 T-cell enumeration and antiretroviral drug toxicity monitoring in primary healthcare clinics using point-of-care testing. *AIDS* 2011; 25: 807–12.
27. Boehme CC, Nicol MP, Nabeta P, et al. Feasibility, diagnostic accuracy, and effectiveness of decentralised use of the Xpert MTB/RIF test for diagnosis of tuberculosis and multidrug resistance: a multicentre implementation study. *Lancet* 2011; 377:1495–505.
28. Boehme CC, Nabeta P, Hillemann D, et al. Rapid molecular detection of tuberculosis and rifampin resistance. *NEJM* 2010; 363:1005–15.
29. Vadwai V, Boehme C, Nabeta P, Shetty A, Alland D, Rodrigues C. Xpert MTB/RIF: a new pillar in diagnosis of extrapulmonary tuberculosis? *Journal of Clinical Microbiology* 2011; 49:2540–5.
30. Rachow A, Zumla A, Heinrich N, et al. Rapid and accurate detection of mycobacterium tuberculosis in sputum samples by Cepheid Xpert MTB/RIF assay—a clinical validation study. *PLoS ONE* 2011; 6:8.
31. Scott LE, McCarthy K, Gous N, et al. Comparison of Xpert MTB/RIF with other nucleic acid technologies for diagnosing pulmonary tuberculosis in a high HIV prevalence setting: a prospective study. *PLoS Med* 2011; 8: e1001061.
32. Lawn SD, Kerkhoff AD, Vogt M, Ghebrekristos Y, Whitelaw A, Wood R. Characteristics and early outcomes of patients with Xpert MTB/RIF-negative pulmonary tuberculosis diagnosed during screening before antiretroviral therapy. *Clinical Infectious Diseases* 2012; 54: 1071–9.
33. Clouse K, Page-Shipp L, Dansey H, et al. Xpert MTB/RIF: Routine point-of-care use at the primary care level. 3rd South African TB Conference, Durban, June 12-15, 2012.
34. World Health Organization. Tuberculosis diagnostics automated DNA test: WHO endorsement and recommendations. Geneva: WHO. Available from: http://www.who.int/tb/features_archive/xpert_factsheet.pdf.
35. Stevens W. GeneXpert implementation in South Africa public sector. Geneva: WHO. Available from: http://www.stoptb.org/wg/gli/assets/html/day 3/Stevens - South Africa.pdf.
36. Gounden V, George J. Multi point of care instrument evaluation for use in anti-retroviral clinics in South Africa. *Clinical Laboratory* 2012; 58: 27–40.
37. Faal M, Naidoo N, Glencross DK, Venter WD, Osih R. Providing immediate CD4 count results at HIV testing improves ART initiation. *J Acquir Immune Defic Syndr* 2011. doi:10.1097/QAI.0b013e3182303921.

38. Larson BA, Bistline K, Ndibongo B, et al. Rapid point-of-care CD4 testing at mobile HIV testing sites to increase linkage to care: An evaluation of a pilot program in South Africa. *J Acquir Immune Defic Syndr* 2012; in press.
39. Myer L, Zulliger R, Black S, Pienaar D, Bekker L-G. Pilot programme for the rapid initiation of antiretroviral therapy in pregnancy in Cape Town, South Africa. A*IDS Care* 2012; : 37–41.
40. Glencross DK, Coetzee LM, Faal M, et al. Performance evaluation of the point-of-care CD4 analyser Pima using capillary blood sampling in field tests in South Africa. *J Int AIDS Soc* 2012; 15: 3.
41. National Department of Health South Africa. Clinical guidelines for the management of HIV & AIDS in adults and adolescents. Pretoria: Department of Health, 2010.
42. Rosen S, Fox MP, Gill CJ. Patient retention in antiretroviral therapy programs in sub-Saharan Africa: a systematic review. *PLoS Med* 2007; 4: e298.
43. Sanne I, Westreich D, Macphail AP, Rubel D, Majuba P, Van Rie A. Long term outcomes of antiretroviral therapy in a large HIV/AIDS care clinic in urban South Africa: a prospective cohort study. *J Int AIDS Soc* 2009; 12: 38.
44. Fox MP, Shearer K, Maskew M, et al. Treatment outcomes after seven years of public-sector HIV treatment at the Themba Lethu Clinic in Johannesburg, South Africa. *AIDS* 2012; in press.

14. Appendices

Appendix 1. Recruiting notice
Appendix 2. Recruiting pamphlet
Appendix 3. Screening form (Track 1 and Track 2)
Appendix 4. Informed consent information sheet and consent form (Track 1 and Track 2)
Appendix 5. Case report form (Track 1 and Track 2)
Appendix 6. Data safety monitoring plan
Appendix 7. Payment voucher information card
Appendix 8. Pre-screening log
Appendix 9. Consent form addendum
